# The interaction between adhesion protein 33 (TvAP33) and BNIP3 mediates the adhesion and pathogenicity of *Trichomonas vaginalis* to host cells

**DOI:** 10.1186/s13071-023-05798-x

**Published:** 2023-06-21

**Authors:** Zhenchao Zhang, Yangyang Deng, Wanxin Sheng, Xiaoxiao Song, Yuhua Li, Fakun Li, Ying Pan, Xiaowei Tian, Zhenke Yang, Shuai Wang, Mingyong Wang, Xuefang Mei

**Affiliations:** 1 Department of Pathogenic Biology, School of Basic Medical Sciences, Xinxiang Medical University, Xinxiang, 453003 Henan People’s Republic of China; 2 Xinxiang Key Laboratory of Pathogenic Biology, School of Basic Medical Sciences, Xinxiang Medical University, Xinxiang, 453003 Henan People’s Republic of China; 3 The Third Affiliated Hospital of Xinxiang Medical University, Xinxiang, 453003 Henan People’s Republic of China; 4 Xinxiang Key Laboratory of Immunoregulation and Molecular Diagnostics, School of Laboratory Medicine, Xinxiang Medical University, Xinxiang, 453003 China; 5 School of Medical Technology, Shangqiu Medical College, Shangqiu, 476100 China

**Keywords:** *T. vaginalis*, Adhesion protein 33, BNIP3, Interaction, Mediate, Pathogenicity

## Abstract

**Background:**

*Trichomonas vaginalis* is a widespread and important sexually transmitted pathogen. Adherence to the surface of the host cell is the precondition for the parasitism and pathogenicity of this parasite.* Trichomonas vaginalis* adhesion protein 33 (TvAP33) plays a key role in the process of adhesion, but how this protein mediates the adhesion and pathogenicity of *T. vaginalis* to host cells is unclear.

**Methods:**

The expression of TvAP33 in trophozoites was knocked down by small interfering RNA. VK2/E6E7 cells and mice infected with *T. vaginalis* were used to evaluate the pathogenicity of *T. vaginalis*. We constructed a complementary DNA library of VK2/E6E7 cells and screened the protein molecules interacting with TvAP33 by the yeast two-hybrid system. The interaction between TvAP33 and BNIP3 (Bcl-2 interacting protein 3) was analyzed by co-immunoprecipitation and colocalization.

**Results:**

Following knockdown of TvAP33 expression, the number of *T. vaginalis* trophozoites adhering to VK2/E6E7 cells decreased significantly, and the inhibition of VK2/E6E7 cell proliferation and VK2/E6E7 cell apoptosis and death induced by *T. vaginalis* were reduced. Animal challenge experiments showed that the pathogenicity of trophozoites decreased following passive immunization with TvAP33 antiserum or blocking of the TvAP33 protein. Immunofluorescence analysis revealed that TvAP33 could bind to VK2/E6E7 cells. Eighteen protein molecules interacting with TvAP33 were identified by the yeast two-hybrid system. The interaction between TvAP33 and BNIP3 was further confirmed by co-immunoprecipitation and colocalization. When the expression of both TvAP33 and BNIP3 in trophozoites was knocked down by small RNA interference, the number of *T. vaginalis* adhering to VK2/E6E7 cells and the inhibition of VK2/E6E7 cell proliferation were significantly lower compared to trophozoites with only knockdown of TvAP33 or only BNIP3. Therefore, the interaction of TvAP33 and BNIP3 in the pathogenesis of *T. vaginalis* infecting host cells is not unique and involves other molecules.

**Conclusions:**

Our study showed that the interaction between TvAP33 and BNIP3 mediated the adhesion and pathogenicity of *T. vaginalis* to host cells, providing a basis for searching for drug targets for *T. vaginalis* as well as new ideas for the prevention and treatment of trichomoniasis.

**Graphical Abstract:**

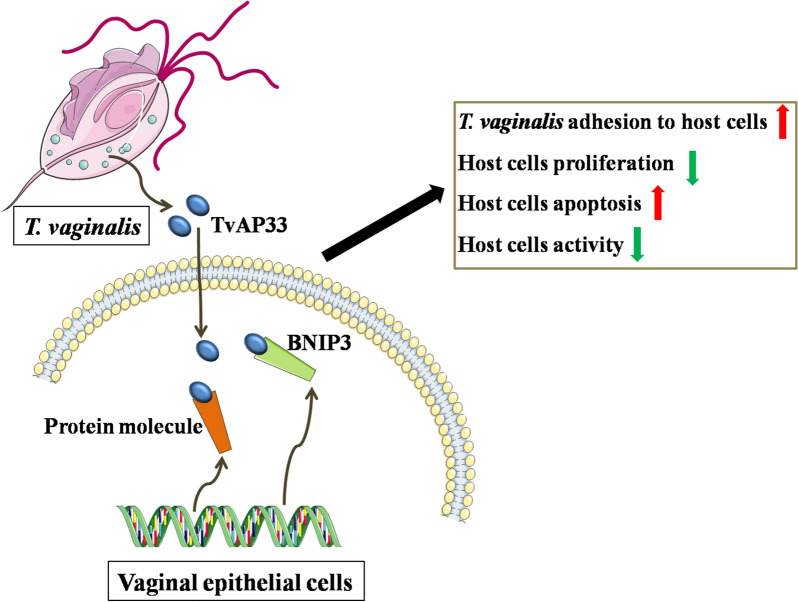

**Supplementary Information:**

The online version contains supplementary material available at 10.1186/s13071-023-05798-x.

## Background

*Trichomonas vaginalis* is a widespread and important sexually transmitted pathogen in humans that causes a common infection of the urogenital system [1]. In its most recent report on *T. vaginalis* infection, the WHO estimated that this infection occurred in 6.3% (95% uncertainty interval [UI] 4.0–7.2) of women and 0.6% (95% UI 0.4–0.9) of men, with regional values ranging from 1.6% to 11.7% in women and from 0.2% to 1.3% in men. Clinical manifestations of *T. vaginalis* infection in women may include vulvovaginal irritation, lower abdominal pain, dyspareunia, dysuria and malodorous vaginal discharge [2, 3]; in contrast, the disease is usually asymptomatic in men although clinical manifestations of dysuria, irritation and urethral discharge may occur in a few cases [4]. In recent years, studies have shown that cervical cancer in women, prostate cancer in men and infertility are associated with *T. vaginalis* infection [5]. Moreover, the widespread prevalence of *T. vaginalis* increases the risk of human infection with human immunodeficiency virus (HIV) and mycoplasma [6, 7]. The high incidence and severe complications of *T. vaginalis* infection support the need for more dynamic approaches to find new drug and vaccine targets.

*Trichomonas vaginalis* is an extracellular parasitic protozoan, adhering to the epithelial lining of the host's urogenital tract to survive. Following the adherence of *T. vaginalis* trophozoites to host cells, the ovoid free-swimming parasite transitions into a more elongated shape with accompanying pseudopodia, which results in the microenvironment of the colonization site of *T. vaginalis* being established. The niches continuously change as a result of different host biological processes and outside forces. Such changes include natural hormonal shifts that could lead to epithelial shedding in the female urogenital tract, excretion that could alter salinity, pH and toxicity of the environment and flux in communities of commensal and infectious microorganisms [8, 9]. However, the morphology of *T. vaginalis* was observed not to change when *T. vaginalis* infected HeLa cells [10], indicating that *T. vaginalis* was able to recognize and specifically bind to host tissues. The adhesion of *T. vaginalis* to different cell types (renal epithelial cells, colon epithelial cells, sperm, fibroblasts, leukocytes or breast myocytes) has been previously reported [11–15]. Therefore, adhesion of these pathogenic organisms to mucosal cells is considered a first and prerequisite step for *T. vaginalis* infections [16, 17]. Okumura et al. found that the galectin-1 expressed by human cervical epithelial cells could bind to lipophosphoglycan on the surface of *T. vaginalis* cells (TvLPG) and that galectin-1 bound to *T. vaginalis* in a carbohydrate-dependent manner that was inhibited in the presence of TvLPG [18].

The adhesion of *T. vaginalis* to host cells was reported to be mainly mediated by five *T. vaginalis* adhesion proteins (TvAPs) on the surface of trophozoites: TvAP23, TvAP33, TvAP51, TvAP65 and TvAP120. Southern blot analysis confirmed the existence of three single-copy TvAP33 genes and suggested a semiconservative genomic arrangement between *T. vaginalis* isolates [19]. By using both antisense inhibition of gene expression and AP33 synthesis and the heterologous expression of AP33 in *Trichomonas foetus*, Mundodi et al. confirmed a role for TvAP33 as an adhesin in *T. vaginalis* [20]. Two domains interacting with host cell surfaces were identified at distinct parts of TvAP33: one in the N-terminal half of the protein and the other within 24 residues in the C-terminal third. Further analysis of the C-terminal binding domain revealed that a peptide representing this area could inhibit *T. vaginalis* cytoadherence by 40% [21]. TvAP33 and TvAP51 are not only localized on the cell surface and mediate *T. vaginalis* cytoadherence but are also characterized as the α- and β-subunits of succinyl-CoA synthetase (SCS) that catalyze key steps of energy metabolism in *T. vaginalis* hydrogenosomes, are biotinylated in the cytosol and then imported exclusively into hydrogenosomes [22]. van der Schee et al. developed a restriction fragment length polymorphism (RFLP) tool for mapping variation in TvAP33 and found that one of the three TvAP33 RFLP types identified appeared to be associated with the absence of *Ureaplasma urealyticum* infection [23]. In our previous research, we found that TvAP33, as a novel antigen, could be used as a protein vaccine against *T. vaginalis* infection [24].

Some researchers believe that TvAPs are controversial adhesion molecules [25]. In the present study, we found that TvAP33 played a role in *T. vaginalis* adherence to host cells, inhibiting the proliferation of host cells and inducing the apoptosis and death of host cells, which was achieved through interaction with receptor molecules on host cells.

## Methods

### Ethics statement

All experiments performed in the present study were reviewed and approved by the Ethics Review Committee of Xinxiang Medical University (Reference No. XYLL-2018S006). We maximized our efforts to alleviate the distress and pain of the experimental animals as much as possible, and the infected mice were euthanized at humane endpoints when the mice appeared moribund. Euthanasia was predominantly performed by placing the experimental animal in a confined space and exposing it to 60–70% CO_2_ for 5 min; occasionally, cervical dislocation was used to confirm the effectiveness of euthanasia.

### Parasites, cells and mice

The strain of *T. vaginalis* used in the present study was maintained in our laboratory and identified as actin genotype E by PCR restriction RFLP, which is the dominant genotype in the city of Xinxiang, Henan Province, China. As described in our previous studies [1, 26], *T. vaginalis* was cultured in complete TYM medium in a humidified chamber maintained at 37 °C and 5% CO_2_. The parasites were collected by centrifugation and used for subsequent research.

Human vaginal epithelial cells (cell line VK2/E6E7) were purchased from American Type Culture Collection (Manassas, VA, USA; ATCC #CRL-2616). The VK2/E6E7 cells were cultured on CnT-Prime Epithelial Cell Culture Medium (Cellntec, Bern, Switzerland) supplemented with 1% penicillin–streptomycin solution, in a humidified chamber at 37 °C and 5% CO_2_. Cells of the 293T cell line were kept in our laboratory and cultured on Dulbecco's Modified Eagle Medium (DMEM) supplemented with 1% penicillin–streptomycin solution and 10% fetal calf serum in a humidified chamber at 37 °C and 5% CO_2_.

Eight-week-old BALB/c mice were purchased from Beijing Vital River Laboratory Animal Technology Co., Ltd. (Beijing, China) and kept under specific pathogen-free conditions.

### Gene cloning and expression of recombinant protein

Total RNA was extracted from *T. vaginalis* trophozoites using the E.Z.N.A.™ Total RNA Kit I (Omega Bio-Tek, Norcross, GA, USA) according to the manufacturer’s instructions, and the genomic DNA was removed using DNase I. Complementary DNA (cDNA) was synthesized by reverse transcription PCR, and then the open reading frames (ORF) of TvAP33 (GenBank accession no. U87098.1) and glyceraldehyde 3-phosphate dehydrogenase (GAPDH; GenBank accession no. L11394.1) were amplified from the cDNA. The TvAP33 and GAPDH fragments were separately cloned into the pET-32a(+) vector to form recombinant plasmids pET-32a-TvAP33 and pET-32a-GAPDH, respectively, for subsequent research. The recombinant plasmids were then sequenced to confirm that the vectors were successfully constructed.

The pET-32a-TvAP33 and pET-32a-GAPDH recombinant plasmids (rpET-32a-TvAP33 and rpET-32a-GAPDH) were introduced into competent *Escherichia coli* BL21(DE3) strain; when the optical density at 600 nm (OD600) of the culture reached 0.6 at 37 °C, isopropyl-b-D-thiogalactopyranoside (IPTG; Sigma–Aldrich, St. Louis, MO, USA) was added to the bacterial growth culture media to induce recombinant protein expression. The rTvAP33 and rGAPDH proteins were then purified by passage through a Ni^2+^-nitrilotriacetic acid (Ni–NTA) column (GE Healthcare, Chicago, IL, USA), and the purified recombinant proteins were determined by 12% (w/v) sodium dodecyl sulfate–polyacrylamide gel electrophoresis (SDS-PAGE). After the concentration was measured, the rTvAP33 and rGAPDH proteins were stored at − 20 °C for future study.

### Antisera against rTvAP33 and rGAPDH proteins

For the generation of polyclonal antibodies (PcAbs), approximately 0.3 mg of the purified rTvAP33 and rGAPDH proteins were mixed with Freund’s complete adjuvant as a 1:1 mixture and subcutaneously injected into SD rats (Beijing Vital River Laboratory Animal Technology Co., Ltd., Beijing, China) in multiple places. Two weeks after the initial injections, a booster was given under the same conditions by using proteins that were mixed with Freund’s incomplete adjuvant as a 1:1 mixture. The rats were then reboosted three times at intervals of 1 week. Finally, the serum was collected, purified and stored until use. Sera collected before the injections of the proteins were used as negative sera.

### RNA interference

According to the gene sequence of TvAP33, three alternative small interfering RNAs (siRNAs) were designed to interfere with the expression of TvAP33 (Table 1). The three alternative siRNAs, negative control siRNA (NC-siRNA) and universal green fluorescein-labeled siRNA, were synthesized by GENEWIZ® (Suzhou, China). Green fluorescein-labeled siRNA was used to optimize the conditions of siRNA transfection into *T. vaginalis* trophozoites; the final concentration of transfected siRNA was set to 0, 50, 100, 150 and 200 nmol, and the transfection time was set to 0, 8, 12, 24 and 36 h.

**Table 1:** The sequences of oligonucleotide primers and small interfering RNAs

Name	Sequence (5′ → 3′)	Description
TvAP33 sense	GGCACATCCGAAGAAGATGC	Oligonucleotide primer sequences used for detecting the mRNA level of TvAP33 by qPCR in this research
TvAP33 antisense	CTTGCCTGGTGGAGCTGTA	
TvActin sense	TCACAGCTCTTGCTCCACCA	
TvActin antisense	AAGCACTTGCGGTGAACGAT	
BNIP3 sense	CAGCATGAGTCTGGACGGAG	Oligonucleotide primer sequences used for detecting the mRNA level of BNIP3 by real-time PCR in this research
BNIP3 antisense	GCCGACTTGACCAATCCCAT	
Actin sense	CGTGACATTAAGGAGAAGCTG	
Actin antisense	CTAGAAGCATTTGCGGTGGAC	
TvAP33-siRNA sense	GUCUCCCAAUCUUCAAGAACATT	The siRNA sequences used for knocking down the expression of TvAP33 in *T. vaginalis* trophozoites
TvAP33-siRNA antisense	UGUUCUUGAAGAUUGGGAGACTT	
TvNC sense	CGUCCCGUAGCCCACUAAAdTdT	
TvNC antisense	UUUAGUGGGCUACGGGACGdTdT	
BNIP3-siRNA sense	GCCUCGGUUUCUAUUUAUAAUTT	The siRNA sequences used for knocking down the expression of BNIP3 in VK2/E6E7 cells
BNIP3-siRNA antisense	AUUAUAAAUAGAAACCGAGGCTT	
NC sense	UUCUCCGAACGUGUCACGUdTdT	
NC antisense	ACGUGACACGUUCGGAGAAdTdT	

The 20 μmol of siRNA and 1.2 μl of Lipofectamine 2000 transfection medium (Invitrogen, Thermo Fisher Scientific, Waltham, MA, USA) were diluted to working concentrations with 50 μl of OptiMEM medium (Thermo Fisher Scientific). The diluted siRNA and Lipofectamine 2000 were mixed evenly to form the transfection complex, which was allowed to stand at room temperature for 20 min. Approximately 5 × 10^5^ T*. vaginalis* trophozoites were inoculated into a 24-well plate, and 100 μl of transfection complex was added to each well. The cell culture plate was placed in a humidified chamber maintained at 37 °C and 5% CO_2_. After 4–6 h, the culture medium containing the transfection complex was replaced by fresh TYM medium, and *T. vaginalis* was cultured for a further 36 h.

### Real-time PCR (qPCR)

According to the gene sequences of TvAP33 and TvActin (GenBank accession no. KF747377.1), primers were designed according to the gene sequences of TvAP33 and TvActin (GenBank accession no. KF747377.1), respectively, and synthesized for detection by real-time PCR (qPCR) (Table 1). The amplification efficiency and specificity of the primers were tested by qPCR with gradient-diluted complementary DNA (cDNA) as a template. The qPCR (RT-PCR) was run on a QuantStudio ^TM^ 5 system (Applied Biosystems, Thermo Fisher Scientific) with SYBR qPCR Master Mix (Vazyme, Nanjing, China). As described in the manufacturer’s instructions, the reaction mixture (20 μl) contained 2 × ChamQ Universal SYBR qPCR Master Mix (10 μl), forward primer (10 μM, 0.4 μl), reverse primer (10 μM, 0.4 μl), cDNA (2 μl) and ddH_2_O (7.2 μl). The amplification protocol consisted of a predenaturation step at 95 °C for 30 s; 40 cycles of a circular reaction at 95 °C 10 s and 60 °C for 30 s; and a melting curve at 95 °C for 15 s, 60 °C for 30 s and 95 °C for 15 s. The messenger RNA (mRNA) expression levels of the target genes, such as TvAP33, were normalized to those of the TvActin primers. Relative expression levels were calculated by the 2^−△△Ct^ method.

### Western blot

*Trichomonas vaginalis* soluble proteins were extracted from trophozoites with siRNA, and the concentration of the proteins was determined by the Bradford method. The soluble proteins of *T. vaginalis* were separated by SDS–PAGE, and the separated proteins were transferred onto nitrocellulose membranes (Merck Chemicals Shanghai Co., Ltd., Shanghai, China). The membrane was blocked with 5% (w/v) skim milk/phosphate buffered saline (PBS)-0.5% Tween 20 (PBST) and then incubated with specific anti-TvAP33 or anti-GAPDH rat antibody (1:100 dilution) for 1 h at 37 °C. The membrane was then washed 3 times with PBST for 5 min, and goat anti-rat immunoglobulin G (IgG)-horseradish peroxidase (Sigma Shanghai, Shanghai, China) was applied as secondary antibodies for 1 h at 37 °C. Finally, an enhanced chemiluminescence (ECL) kit (Vazyme Biotech, Nanjing, China) was used to visualize the bands on the basis of the operating manuals.

### Detection of *T. vaginalis* adhesion to VK2/E6E7 cells

*Trichomonas vaginalis* trophozoites were treated with TvAP33-siRNA and NC-siRNA and divided into the TvAP33 group and NC group, respectively. *Trichomonas vaginalis* without siRNA treatment was used as the *T. vaginalis* group. Three parallel controls were set in each group (*n* = 3). VK2/E6E7 cells were inoculated into a 24-well plate containing cell climbing slices. When the growth convergence of cells reached 80–90%, *T. vaginalis* in each group was labeled with CytoTrace™ Orange (Sigma-Aldrich, St. Louis, MO, USA) and then used to infect VK2/E6E7 cells at a ratio of 1:1 (parasite:cell) for 15 and 30 min. The cell climbing slices were then fixed with 4% paraformaldehyde for 10 min at room temperature in the dark and subsequently examined by fluorescence microscopy (Nikon Corp., Tokyo, Japan). Five visual fields were randomly selected for each sample, and the number of trophozoites and cells was counted. In each field of vision, the adherence rate (in percentage) of *T. vaginalis* to host cells was calculated as follows: (the number of *T. vaginalis*/the number of VK2/E6E7 cells) × 100. The analyses were conducted based on data obtained from three individual experiments.

### Cell proliferation analysis by MTT assay

VK2/E6E7 cells (1 × 10^5^ cells/well) were inoculated into a 96-well plate. *Trichomonas vaginalis* trophozoites were treated with TvAP33-siRNA and NC-siRNA and divided into the TvAP33 group and NC group. Untreated *T. vaginalis* and uninfected VK2/E6E7 cells were used as the *T. vaginalis* group and blank group, respectively. The VK2/E6E7 cells were infected with *T. vaginalis* at a ratio of 1:1 (parasite:cell) for 12 h. Five parallel controls were used in each group (*n* = 5). After MTT solution (5 mg/ml) had been added to each well (20 μl/well), the VK2/E6E7 cells were cultured for a further 4 h at 37 °C and 5% CO_2_. The liquid and *T. vaginalis* were removed and the culture plates washed 3 times with PBS, following which 150 μl DMSO was added to each well, and the 96-well plate was shaken slightly for 10 min. Light absorption at 570 nm was measured on a microplate reader (MULTISKAN FC; Thermo Fisher Scientific). The cell proliferation index was calculated as follows: (the OD value of the *T. vaginalis*-infected group − the OD value of the cell culture plate)/(the OD value of the blank group − the OD value of the cell culture plate). The analyses were conducted based on data obtained from three individual experiments.

### Analysis of cell apoptosis

As described above, the VK2/E6E7 cells were infected with *T. vaginalis* trophozoites at a ratio of 1:1 (parasite:cell) for 12 h under conditions of 37 °C and 5% CO_2_, and then divided into the TvAP33 group, NC group and *T. vaginalis* group. Three parallel controls were set in each group (*n* = 3). The VK2/E6E7 cells were separated from the 24-well plate by trypsin and then resuspended in 500 μl of binding buffer after being washed twice with PBS by centrifugation at 500 *g* for 5 min. The supernatant was removed by centrifugation and resuspended in 100 μl of binding buffer. A 5-μl aliquot of Annexin V-FITC reagent (Sigma-Aldrich) and 5 μl of propidium iodide (PI) were added to the cell suspension, which was mixed and incubated at room temperature in the dark for 5 min. Samples of the cell suspension were measured by flow cytometry within 1 h. The independent experiment was repeated 3 times.

### Cell activity test

As described above, the VK2/E6E7 cells were infected with *T. vaginalis* trophozoites at a ratio of 1:1 (parasite:cell) for 12 h under conditions of 37 °C and 5% CO_2_, and then divided into the TvAP33 group, NC group, *T. vaginalis* group and blank group. Five parallel controls were set in each group (*n* = 5). The cell culture medium and *T. vaginalis* were removed and the culture plates washed 2 times with PBS. PBS and 0.4% trypan blue were then added at a ratio of 9:1, and the cells were examined by optical microscopy (Nikon Corp.). Five visual fields were randomly selected for each sample, and the number of cells was counted. In each field of vision, the percentage cell viability was calculated as follows: (the number of unstained cells/the number of total cells) × 100. The analyses were conducted based on data obtained from three individual experiments.

### Animal challenge against *T. vaginalis*

#### Passive immunization with anti-rTvAP33 PcAb

Forty-five BALB/c mice aged 6 weeks were randomly divided into three groups (*n* = 15), and each mouse was intraperitoneally immunized with 200 μl of anti-rTvAP33 PcAb (200 μg/ml), rat normal IgG (200 μg/ml) or PBS. Mice in each group were immunized 4 times, with a 1-day interval between injections, and each mouse was intraperitoneally infected with 1 × 10^7^ *T. vaginalis* trophozoites after the first immunization. The survival of the mice was monitored throughout the month after the challenge, and animals showing symptoms euthanized by CO_2_. Thirty days later, the following formula was used to calculate the percentage survival rate of mice challenged with *T. vaginalis*: the number of surviving mice/the number of mice before immunization × 100.

#### Antiserum blocking TvAP33 protein

Forty-five BALB/c mice aged 6 weeks were randomly divided into three groups (*n* = 15). *Trichomonas vaginalis* trophozoites were blocked in anti-rTvAP33 PcAb diluted in PBS (anti-rTvAP65 PcAb:PBS = 1:5) at 37 °C for 15 min. Under the same conditions, the trophozoites of the other two groups were treated with normal rat IgG or PBS. The mice were intraperitoneally infected with blocked *T. vaginalis* trophozoites (1 × 10^7^ trophozoites/mouse). The survival of mice was monitored throughout the month after the challenge, and animals showing symptoms were euthanized by CO_2_. Thirty days later, the following formula was used to calculate the percentage survival rate of mice challenged with *T. vaginalis*: the number of surviving mice/the number of mice before immunization × 100.

### Immunofluorescence analysis of TvAP33 binding to host cells

VK2/E6E7 cells were inoculated into a 24-well plate containing cell climbing slices. When the growth convergence of cells reached 90%, the cells were cocultured with the final concentration of 20 μg/ml of the TvAP33 recombinant proteins at 37 °C and 5% CO_2_ for 30 min; the same concentration of pET-32a protein was used as the control. After three washes in PBS, the VK2/E6E7 cells were fixed with 4% paraformaldehyde in Tris-buffered saline (TBS) for 10 min at room temperature, permeabilized with 1% TritonX-100 in TBS for 10 min, washed 3 times in TBS containing 0.05% Tween-20 (TBST) and blocked with TBST containing 5% (w/v) bovine serum albumin (BSA) for 1 h at 37 °C. After three washes in TBST, TvAP65 antiserum (1:100 dilution) was added and to the well and incubated at 4 °C overnight. After a further three washes in TBST, the cells were maintained in darkness for 40 min in goat anti-rat IgG antibody (Beyotime, Shanghai, China) labeled with Cy3 diluted at 1:1000. After three washes in PBS, the fluorescent stain 4′,6-diamidino-2-phenylindole (DAPI; Beyotime) was used to stain the nuclei for 15 min in darkness. After washing with TBST, fluorescent mounting medium (Beyotime) was added, and the cells were examined by fluorescence microscopy (Nikon Corp.).

### Construction of the bait vector and cDNA library of VK2/E6E7 cells

The ORF of TvAP33 was cloned and inserted into the bait vector pDHB1 to form pDHB1-TvAP33. The cDNA library of VK2/E6E7 cells was constructed according to the instructions of the SMART cDNA Library Construction Kit (TaKaRa, Clontech Laboratories, CA, USA). In brief, RNase-free DNase I (TaKaRa, Clontech Laboratories) was used to remove the genomic DNA contamination from the prepared RNA samples. The mRNA was then isolated using the Oligotex mRNA Midi Kit (Qiagen, Hilden, Germany). The purified mRNA was used to synthesize double-stranded cDNA, and both ends of the cDNAs were equipped with connectors for recombination into the prey vector. The cDNAs were purified and cloned into the pray plasmid pPR3-N with the BP Clonase^®^ II enzyme (Thermo Fisher Scientific). These recombinant plasmids were electroporated into *E. coli* DH10B, according to the following electrotransfer procedure: voltage 1500 V, resistance 200 Ω and capacity 25 μF. These bacterial solutions were diluted and coated onto solid medium, the number of bacterial clones was counted and the capacity of the library was calculated. The plasmid DNA was randomly extracted from 24 clones and restriction digested by *Sfi*I to confirm the size of the inserts in the clones.

### Identification of binding partners for TvAP33 by the yeast two-hybrid system

The DUALhunter starter kit (Dualsystems Biotech AG, Schlieren, Switzerland) was used to identify the binding molecule of TvAP33 from VK2/E6E7 cells. The bait plasmid pDHB1-TvAP33 was transformed into yeast NMY51. After confirmation of the expression of the bait and functional assay and optimization of the screening stringency, the plasmid pDHB1-TvAP33 was used to screen the proteins interacting with TvAP33 from the VK2/E6E7 cell cDNA library. Positive colonies were picked, and the plasmids were extracted by using the Yeast Plasmid Extraction Kit (Omega Bio-Tek). The selected prey plasmids were transformed into *E. coli* DH5α for amplification. The primers pPR3N-F and pPR3N-R were used to detect the gene fragments inserted in the selected prey plasmids by PCR. The isolated positive prey plasmids were retransformed into yeast NMY51 which contained the bait plasmid pDHB1-TvAP33 to verify the interaction between the two molecules. pTSU2-APP was used as a bait control, and pPR3-N or pNubG-Fe65 was used as the negative or positive prey control, respectively. The fragments inserted in these prey plasmids were sequenced, and the DNA sequences were matched in GenBank to obtain genetic information.

### Analysis of the interaction between TvAP33 and Bcl-2 interacting protein 3

#### Co-immunoprecipitation

The eukaryotic expression vectors pDsRed-N1-TvAP33 and pCMV-3HA-BNIP3 (Bcl-2 interacting protein 3) were successfully constructed and cotransferred into 293 T cells (60 mm well, 12 μl Lipofectamine 2000). After 48 h, the cells were washed with PBS and harvested. Co-immunoprecipitation was performed by using the Pierce™ Co-Immunoprecipitation Kit (Thermo Fisher Scientific) according to the manufacturer's instructions. In brief, the AminoLink Plus Coupling Resin was immobilized by 3.5 μg of affinity-purified anti-Flag antibody or mouse IgG as control (Thermo Fisher Scientific) at 37 °C for 2 h. Then, 350 μl of ice-cold IP Lysis Buffer and protein mixture (FLAG-TvAP33 and HA-BNIP3) from one 60-mm plate of transfected 293 T cells was added to immobilization resin and gently mixed for 2 h at room temperature. After washing, the bound samples were eluted in 60 μl of elution buffer via centrifugation at 2000 *g* for 2 min, following which the protein samples were analyzed by western blot.

#### Immunocolocalization

As described above, the colocalization of TvAP33 and BNIP3 was analyzed by immunofluorescence. The primary antibodies anti-TvAP33 and anti-BNIP3 were used to recognize TvAP33 and BNIP3, respectively.

### Detection of the effect of TvAP33 and BNIP3 on *T. vaginalis* infection of VK2/E6E7 cells

The expression of BNIP3 was knocked down in VK2/E6E7 cells as described above. VK2/E6E7 cells with low expression of BNIP3 were infected with *T. vaginalis* trophozoites with knocked down TvAP33 expression, and then the adhesion of *T. vaginalis* to VK2/E6E7 cells, cell proliferation and cell activity were detected as mentioned above.

### Statistical analyses

One-way analysis of variance (ANOVA) followed by Duncan’s multiple range test was performed to analyze the differences among different experimental groups. The survival-related data were analyzed by the Kaplan–Meier method. SPSS for Windows version 16 software (IBM Corp., Armonk, NY, USA) was used for the statistical analyses, and *P* < 0.05 indicated statistical significance.

## Results

### Knockdown of the expression of TvAP33 in *T. vaginalis* trophozoites

**Fig. 1:**
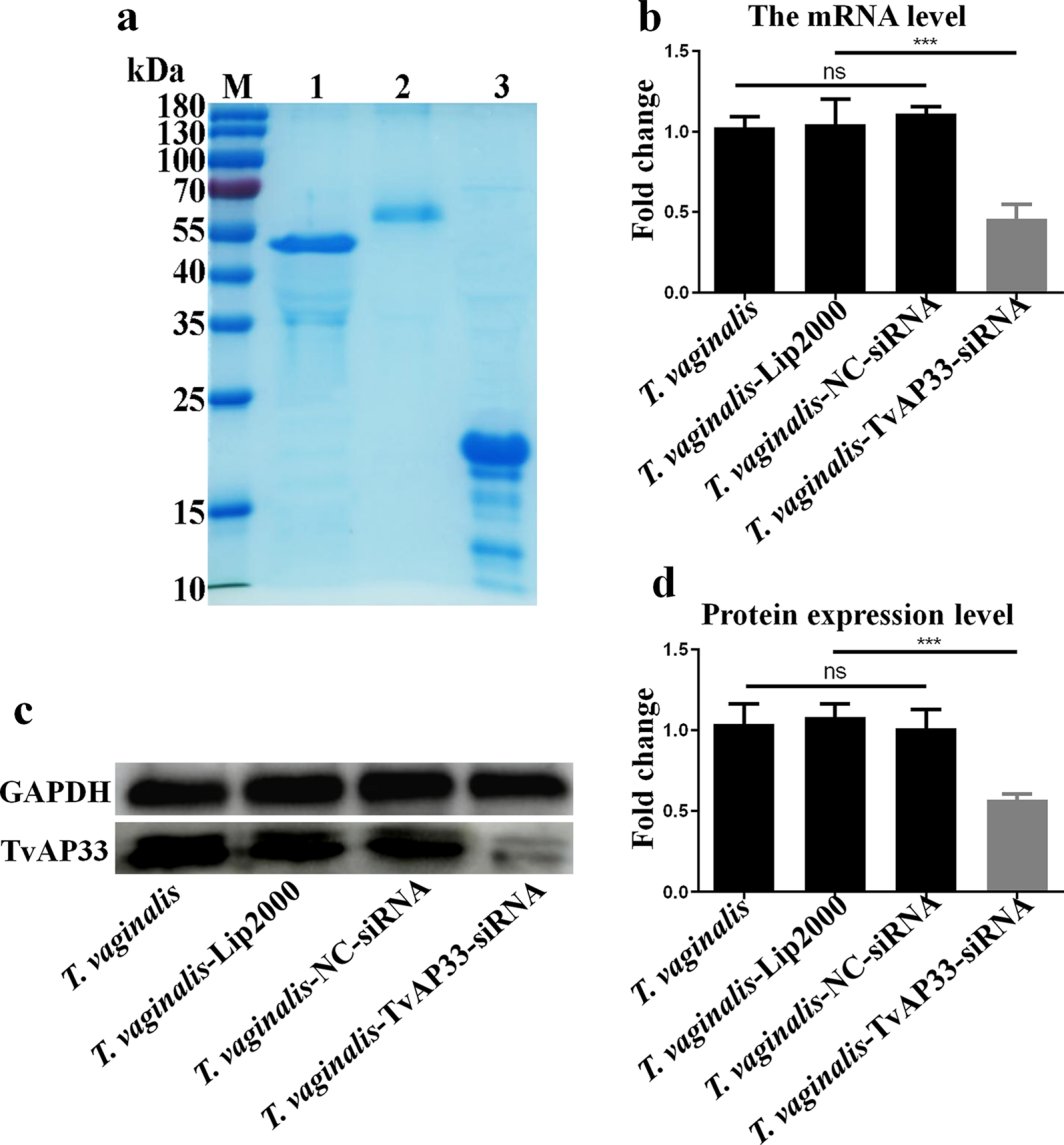
The expression of TvAP33 was knocked down by siRNA in *Trichomonas vaginalis* trophozoites. **a** Sodium dodecyl sulfate-polyacrylamide gel electrophoresis of purified recombinant TvAP33, GAPDH and pET-32a protein. Lanes:* M* Protein marker (values are in kDa),* 1* recombinant TvAP33 protein,* 2* recombinant GAPDH protein,* 3* recombinant pET-32a protein. **b** Messenger RNA level of TvAP33. **c** Protein level of TvAP33. **d** Histogram showing the relative expression levels of TvAP33 as measured in three independent experiments. *p* ≥ 0.05 and *p* < 0.001 represent statistical significances and was labeled as “ns” and “***”, respectively

We successfully constructed the prokaryotic expression vectors pET-32a-TvAP33 and pET-32a-GAPDH and expressed and purified the recombinant proteins TvAP33 and GAPDH (Fig. 1a) to prepare the PcAbs of anti-rTvAP33 and anti-rGAPDH, which were used to detect the changes in TvAP33 expression in *T. vaginalis* trophozoites. The primers detecting the mRNA level of TvAP33 and the siRNA interfering with TvAP33 expression were screened by qPCR (Additional file 1: Figure S1; Additional file 2: Figure S2). By transfecting siRNA labeled with fluorescent dye to optimize the transfection conditions, we found that the optimal duration and siRNA concentration were 36 h and 200 nM, respectively, for transfecting siRNA into *T. vaginalis* trophozoites (Additional file 3: Figure S3). Under the optimized conditions, siRNA interference was completed, and qPCR and western blot analyses showed that the mRNA level and protein expression of TvAP33 were successfully knocked down by TvAP33-siRNA in *T. vaginalis* trophozoites (Fig. 1b–d).

### Effect of TvAP33 on the infection of VK2/E6E7 cells by* T. vaginalis*

**Fig. 2:**
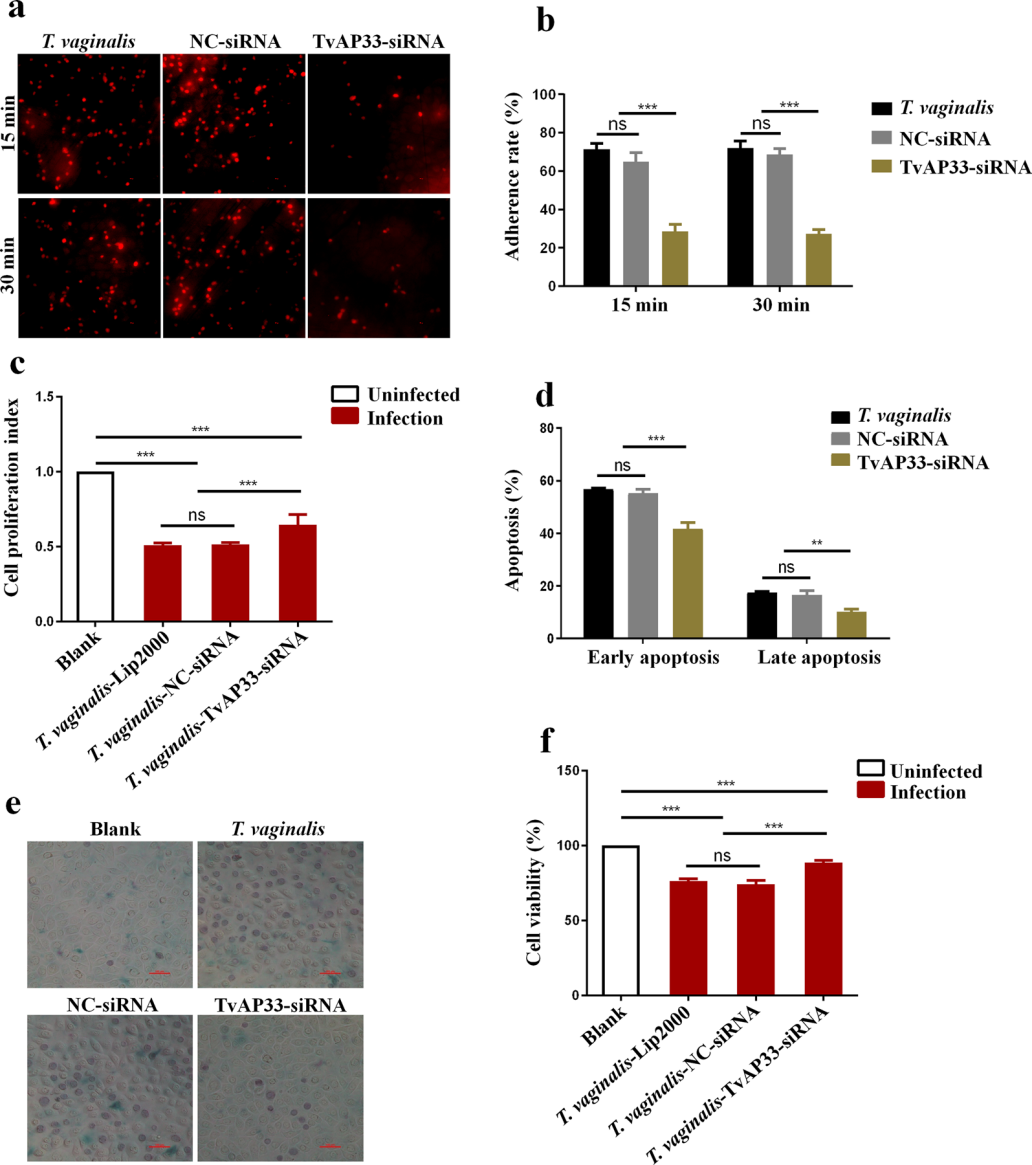
Effect of TvAP33 on the infection of VK2/E6E7 cells by* Trichomonas vaginalis.* **a** Observation of *T. vaginalis* adherence to VK2/E6E7 cells under the fluorescence microscope (40×). The trophozoites were stained with red fluorescence by CytoTrace™ Orange. **b** Statistical analysis of the adherence rate of *T. vaginalis* on VK2/E6E7 cells. After *T. vaginalis* infection for 15 min and 30 min, the adhesion rate of trophozoites on VK2/E6E7 cells in the TvAP33 group was significantly lower than that on cells of the *T. vaginalis* group and the NC group. **c** Analysis of the proliferation of VK2/E6E7 cell by the MTT assay after VK2/E6E7 cells were infected with *T. vaginalis.* The proliferation index of VK2/E6E7 cells in the TvAP33 group was significantly higher than that of cells in the *T. vaginalis* group and NC group. **d** Detection of VK2/E6E7 cell apoptosis after VK2/E6E7 cells were infected with *T. vaginalis.* The early and late apoptosis rates of VK2/E6E7 cells in the TvAP33 group were significantly lower than those of the cells in the *T. vaginalis* group and the NC group. **e** Observation of VK2/E6E7 cell viability under the microscope (40×). The dead cells were stained with blue by trypan blue. **f** Statistical analysis of VK2/E6E7 cell viability after VK2/E6E7 cells were infected with *T. vaginalis.* VK2/E6E7 cell viability in the TvAP33 group was significantly increased, compared with cell viability in the *T. vaginalis* group and the NC group. The results of adherence rate, cell proliferation index, apoptosis and cell viability are presented as the mean ± SD. *p* ≥0.05, *p* <0.05, *p* <0.01 and *p* <0.001 represent statistical significances and was labeled as “ns”, “*”, “**” and “***”, respectively

As shown in Fig. 2a, b, trophozoites (red fluorescence) adhering to VK2/E6E7 cells were observed and counted under a fluorescence microscope. After *T. vaginalis* infection for 15 min, in the TvAP33 group, the adhesion rate of trophozoites on VK2/E6E7 cells (28.60 ± 3.58%) was significantly lower than that on cells the *T. vaginalis* group (71.00 ± 3.81%) and the NC group (67.00 ± 3.54%). Similarly, after *T. vaginalis* infection for 30 min, compared with the adhesion rate of the *T. vaginalis* group (73.20 ± 4.32%) and the NC group (68.80 ± 2.55%), the adhesion rate in the TvAP33 group (27.40 ± 2.07%) was significantly reduced. These results indicate that TvAP33 played an important role in the adhesion of *T. vaginalis* to host cells.

We detected the proliferation of VK2/E6E7 cells by MTT assays (Fig. 2c), and the result showed that after VK2/E6E7 cells were infected with *T. vaginalis*, the proliferation index of VK2/E6E7 cells (0.65 ± 0.07) in the TvAP33 group was significantly higher than that of cells in the *T. vaginalis* group (0.51 ± 0.01) and NC group (0.51 ± 0.01). Flow cytometry detection of apoptosis (Fig. 2d) showed that the early and late apoptosis rates of VK2/E6E7 cells in the TvAP33 group (41.74 ± 2.30% and 10.26 ± 1.00%, respectively) was significantly lower than those of cells in the *T. vaginalis* group (56.76 ± 0.51% and 16.27 ± 2.28%, respectively) and the NC group (55.19 ± 1.51% and 16.61 ± 1.53%, respectively). Under the microscope (Fig. 2e, f), we observed that VK2/E6E7 cell viability in the TvAP33 group (88.50 ± 1.59%) was significantly increased compared with that in the *T. vaginalis* group (76.34 ± 1.50%) and the NC group (74.08 ± 2.60%). These results showed that TvAP33 could inhibit cell proliferation and promote cell apoptosis and death during the process of *T. vaginalis* infecting host cells.

### Evaluating the pathogenicity of trophozoites after TvAP33 blockade

**Fig. 3:**
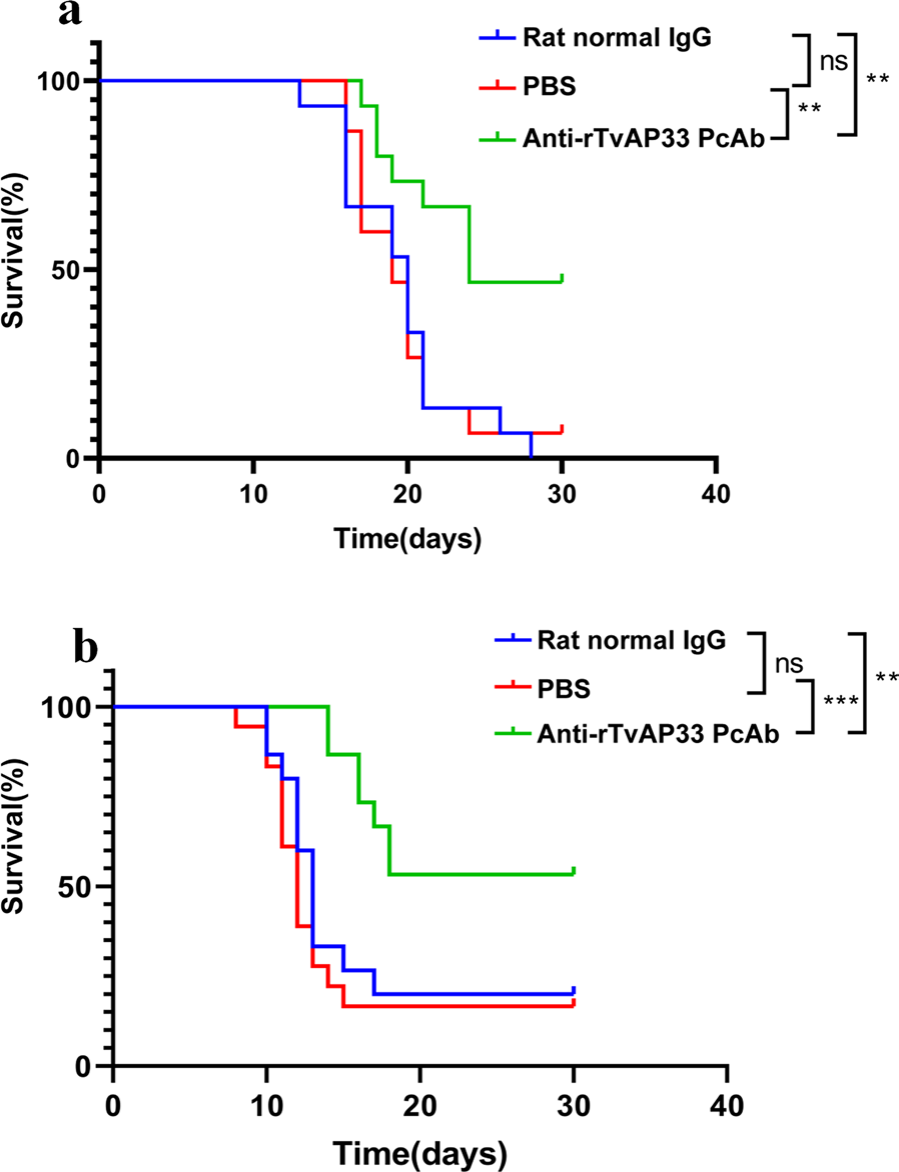
Survival curve of mice after challenge infection with *Trichomonas vaginalis* trophozoites. **a** Mice were challenged with *T. vaginalis* (1 × 10^7^ trophozoites/mouse), after passively immunized with anti-rTvAP33 PcAb. **b** Mice were challenged with *T. vaginalis* (1 × 10^7^ trophozoites/mouse) blocked by anti-rTvAP33 PcAb. *p* ≥ 0.05, *p* < 0.05, *p* < 0.01 and *p* < 0.001 represent statistical significances and was labeled as "ns", "*", "**" and "***", respectively

The survival rate of the different groups of mice challenged with *T. vaginalis* was recorded (Fig. 3a) after mice were passively immunized with the PcAb. The survival rate of the mice in the passively immunized anti-rTvAP33 PcAb group was significantly higher than that of the rats in the normal IgG and PBS groups. In the rat normal IgG and PBS groups, most mice (90–100%) died within 28 days. The survival time of mice in the anti-rTvAP33 PcAb group was (25.00 ± 5.29 days), which was significantly longer than that in the rat normal IgG group (18.47 ± 3.91 days) and PBS group (18.67 ± 3.85 days).

Similarly, as shown in Fig. 3b, compared with the mice infected with *T. vaginalis* blocked by rat normal IgG (15.27 ± 7.82 days) and PBS (10.60 ± 1.64 days), the survival rate of the mice infected with *T. vaginalis* blocked by anti-rTvAP33 PcAb (53.33%) was significantly increased, and the survival time (23.53 ± 7.24 days) was significantly prolonged.

These results suggested that the pathogenicity of *T. vaginalis* was weakened after TvAP33 was blocked.

### Identification of proteins interacting with TvAP33 on VK2/E6E7 cells

**Fig. 4:**
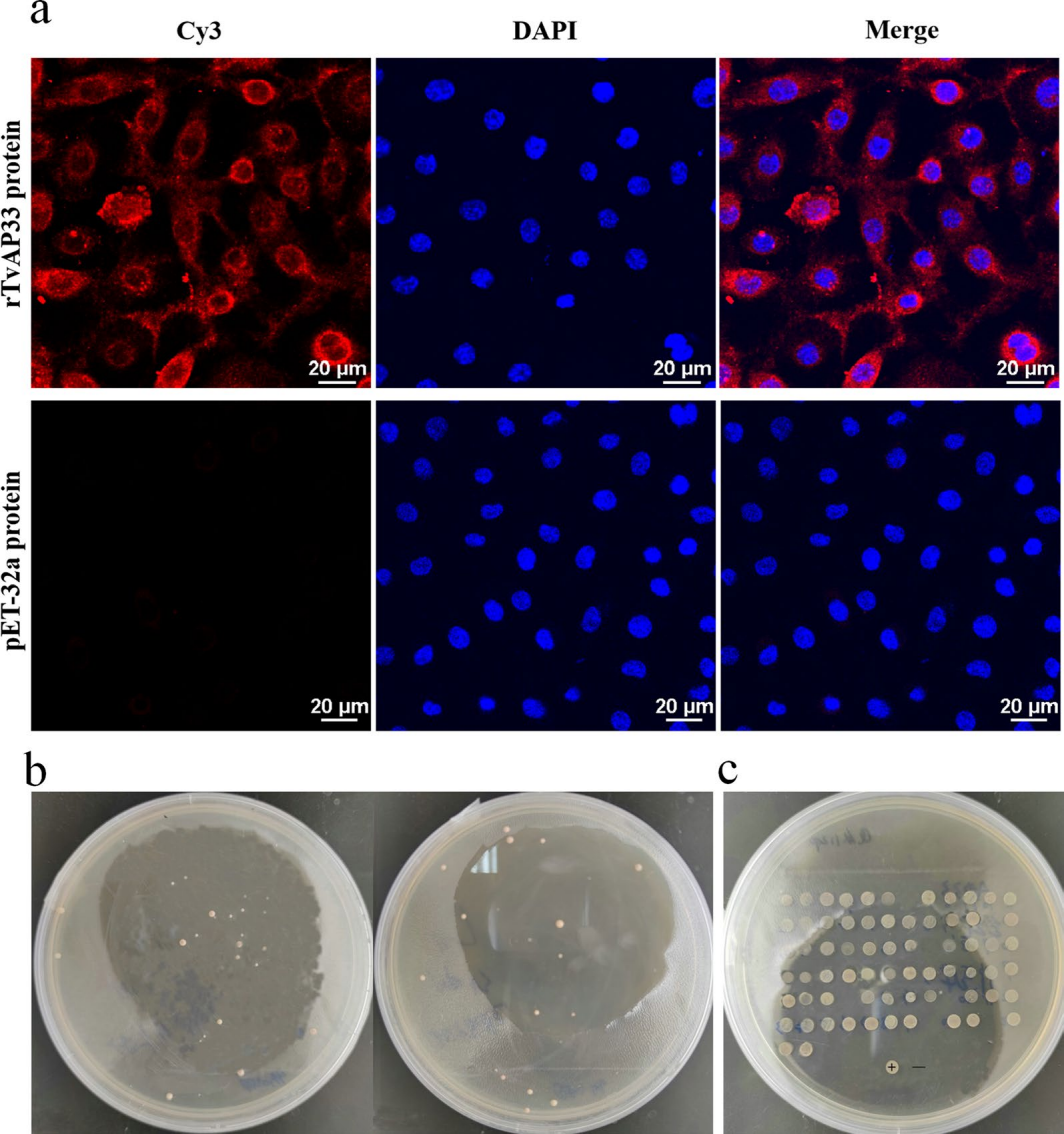
Interaction between TvAP33 protein and molecules in host cells. **a** Immunofluorescence assay of TvAP33 binding to VK2/E6E7 cells. The VK2/E6E7 cells were cocultured with the final concentration of 20 μg/ml of rTvAP33 at 37 °C and 5% CO_2_ for 30 min, and the same concentration of pET-32a protein was used as the control. Cy3 dye (red) was used to label TvAP33 protein bound to VK2/E6E7 cells and DAPI (blue) was used to stain the nuclei. **b** Preliminary screening of molecules interacting with TvAP33 on VK2/E6E7 cells by the yeast two-hybrid system. **c** Second screening by yeast two-hybrid

After VK2/E6E7 cells were cocultured with rTvAP33, we found that rTvAP33 (red fluorescence) could bind to VK2/E6E7 cells (Fig. 4a), which suggested that TvAP33 could adhere to host cells and interact with molecules on host cells. No red fluorescence was observed in the pET-32a protein control group.

We successfully constructed the cDNA library of VK2/E6E7 cells by the gateway method (Additional file 4: Figure S4). The identification results of the primary library indicated that the recombination efficiency was 96%, the average insert size was > 1.0 kb and the cDNA library contained at least 1.04 × 10^7^ primary recombinants. The secondary library was obtained by recombining the plasmid of the primary library, and the recombination efficiency was 100%. In the secondary library, the average insert size was also > 1.0 kb, and the cDNA library contained 1.36 × 10^7^ secondary recombinants. All metrics of this cDNA library conformed to the requirements, and this cDNA library could be used for subsequent screening.

The functionality and self-activation of the yeast two-hybrid system were tested, and the results showed that the bait pDHB1-TvAP33 and the screening system could be used to capture the molecules interacting with TvAP33 from the cDNA library of VK2/E6E7 cells (Additional file 5: Figure S5). A total of 74 positive clones encoding proteins that showed a potential interaction with TvAP33 in yeast cells were obtained in the the yeast two-hybrid analysis (Fig. 4b, c). The screened molecules were analyzed by DNA sequencing and a searching of GenBank; 26 proteins interacting with TvAP33 were identified after removing the molecules screened repeatedly. These 26 molecules were cotransformed into yeast cells with bait TvAP33 to verify the interaction, and 18 of the 26 proteins were identified again (Table 2; Additional file 6: Figure S6). BNIP3, as a multifunctional protein, was selected for subsequent studies.

**Table 2:** Gene information of* Trichomonas vaginalis* adhesion protein 33 interacting molecules screened by yeast two-hybrid assay

Interaction protein number	Name of interacting protein	GenBank accession no	Frequency screened by yeast two-hybrid assay
1	Sterol-4-alpha-carboxylate 3-dehydrogenase, decarboxylating isoform X1 [*Homo sapiens*]	XP_016885053.1	1
2	BNIP3 protein, partial [*Homo sapiens*]	AAH80643.1	1
3	heme oxygenase 2 isoform b [Homo sapiens]	NP_001120676.1	2
4	ER membrane protein complex subunit 6 [*Homo sapiens*]	NP_001014764.1	1
5	CD63 antigen isoform A [*Homo sapiens*]	NP_001244318.1	2
6	transmembrane protein 134 isoform a [*Homo sapiens*]	NP_079400.1	1
7	cytochrome b5 isoform 1 [*Homo sapiens*]	NP_683725.1	2
8	transmembrane protein 147 isoform 1 [*Homo sapiens*]	NP_116024.1	1
9	Solute carrier family 25 member 3 isoform b precursor variant [*Homo sapiens*]	BAD96378.1	1
10	Unnamed protein product [*Homo sapiens*]	BAC11187.1	1
11	V-type proton ATPase 21 kDa proteolipid subunit isoform 1 [Homo sapiens]	NP_004038.1	1
12	Solution structure of human mitochondria fission protein Fis1 [*Homo sapiens*]	1PC2_A	2
13	Protein ABHD14A [Homo sapiens]	NP_056222.2	1
14	*Homo sapiens* cDNA FLJ40371 fis, clone TESTI2034931, highly similar to Cofilin-1	AK097690.1	1
15	p53 apoptosis effector related to PMP-22 [Homo sapiens]	NP_071404.2	1
16	Radiation-inducible immediate early response gene IEX1 [Homo sapiens]	AAC33793.1	1
17	SLC25A3 protein [Homo sapiens]	AAH15379.2	1
18	Uncharacterized protein C4orf3 isoform 1 [Homo sapiens]	NP_001163801.1	1

### Co-immunoprecipitation and immunocolocalization to verify the interaction between TvAP33 and BNIP3

**Fig. 5:**
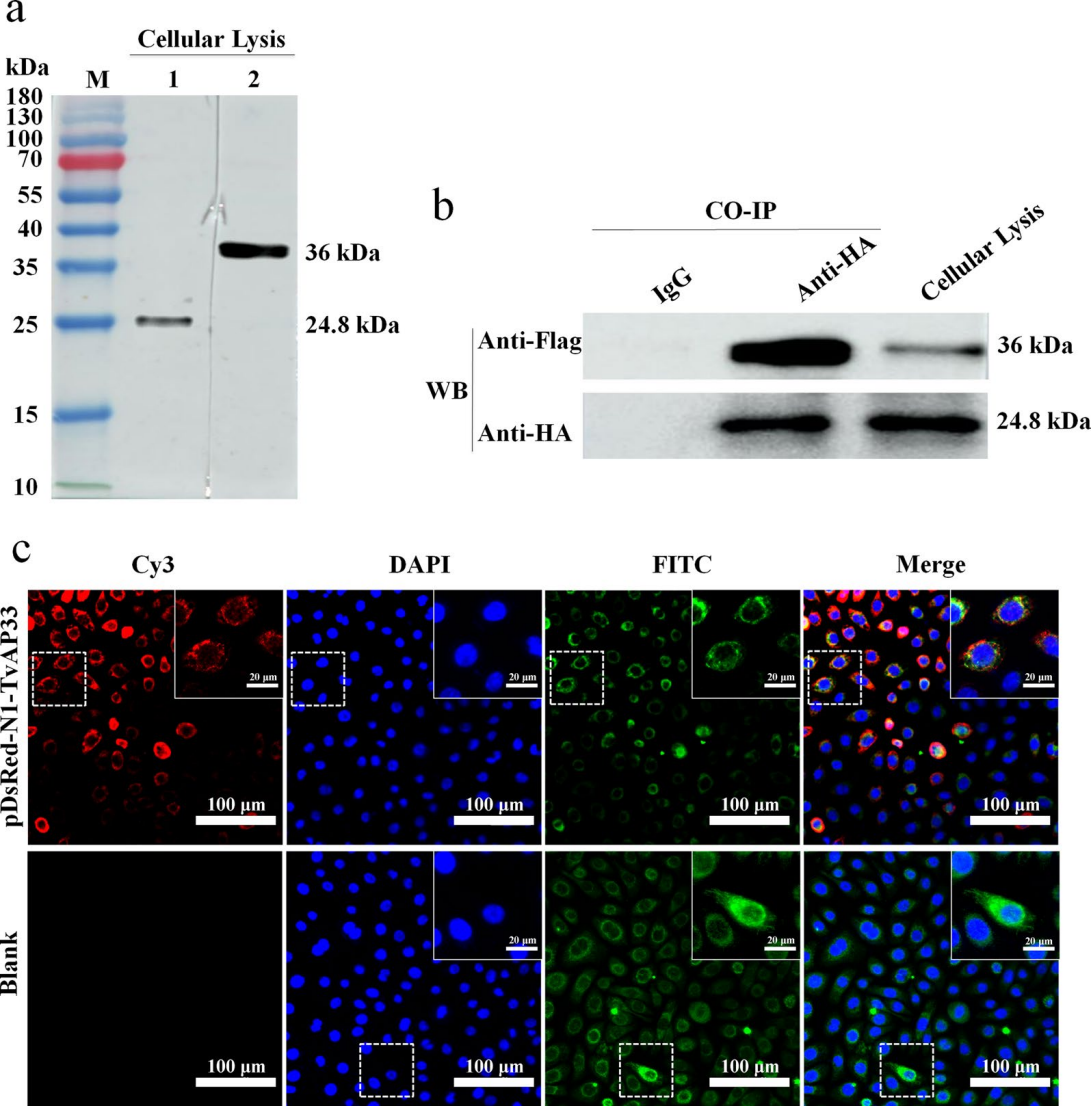
Co-immunoprecipitation and immunocolocalization to analyze the interaction between TvAP33 and BNIP3. **a** Western blot analysis of the expression of TvAP33 and BNIP3 in 293 T cells. Lanes:* M* Protein molecular weight marker (ordinate values in kDa),* 1* after transfection of 293 T cells with pCMV-3HA-BNIP3, the primary antibody of anti-BNIP3 was used to recognize the expression of BNIP3 in cells,* 2* after transfection of 293 T cells with pDsRed-N1-TvAP33, the primary antibody of anti-TvAP33 was used to recognize the expression of TvAP33 in cells. **b** Co-immunoprecipitation to verify the interaction between TvAP33 and BNIP3. After cotransfection of 293 T cells with pDsRed-N1-TvAP33 and pCMV-3HA-BNIP3, the immune precipitates were obtained from cellular lysis by the AminoLink Plus Coupling Resin immobilized with affinity-purified anti-Flag antibody or mouse IgG as control. The primary antibodies of anti-Flag and anti-HA were used to confirm TvAP33 and BNIP3 in precipitates and cellular lysis, respectively. **c** Immunocolocalization to verify the interaction between TvAP33 and BNIP3 in VK2/E6E7 cells. After transfection of VK2/E6E7 cells with pDsRed-N1-TvAP33, the primary antibodies of anti-Flag and anti-BNIP3 were used to confirm TvAP33 and BNIP3 in VK2/E6E7 cells, respectively. The Cy3 (red) and FITC (green) was used to label TvAP33 and BNIP3, and the DAPI (blue) was used to stain the nuclei. Blank VK2/E6E7 cells were used as controls

We successfully constructed the eukaryotic expression vectors pDsRed-N1-TvAP33 and pCMV-3HA-BNIP3, which expressed rTvAP33 and rBNIP3 fused with Flag and HA tag proteins, respectively. The two expression vectors were cotransfected into 293 T cells, and the expression of TvAP65 and BNIP3 in cells was analyzed by western blot. The results showed that the anti-TvAP33 and anti-BNIP3 antibodies could recognize both TvAP33 and BNIP3 expressed in 293 T cells (Fig. 5a). As shown in Fig. 5b, the interaction between TvAP33 and BNIP3 in cells was detected by co-immunoprecipitation, and the results further confirmed that there was an interaction between TvAP33 and BNIP3.

The eukaryotic expression vector pDsRed-N1-TvAP33 was transfected into VK2/E6E7 cells, and the interaction and localization between TvAP33 and BNIP3 were analyzed by immunofluorescence (Fig. 5c). Under a fluorescence microscope, we observed that part of TvAP33 could colocalize with BNIP3, indicating that TvAP33 interacted with other molecules in VK2/E6E7 cells in addition to BNIP3, which was consistent with the results of the screening of multiple interacting molecules from the cDNA library of VK2/E6E7 cells in the yeast two-hybrid assays.

### Knockdown of BNIP3 expression in VK2/E6E7 cells

**Fig. 6:**
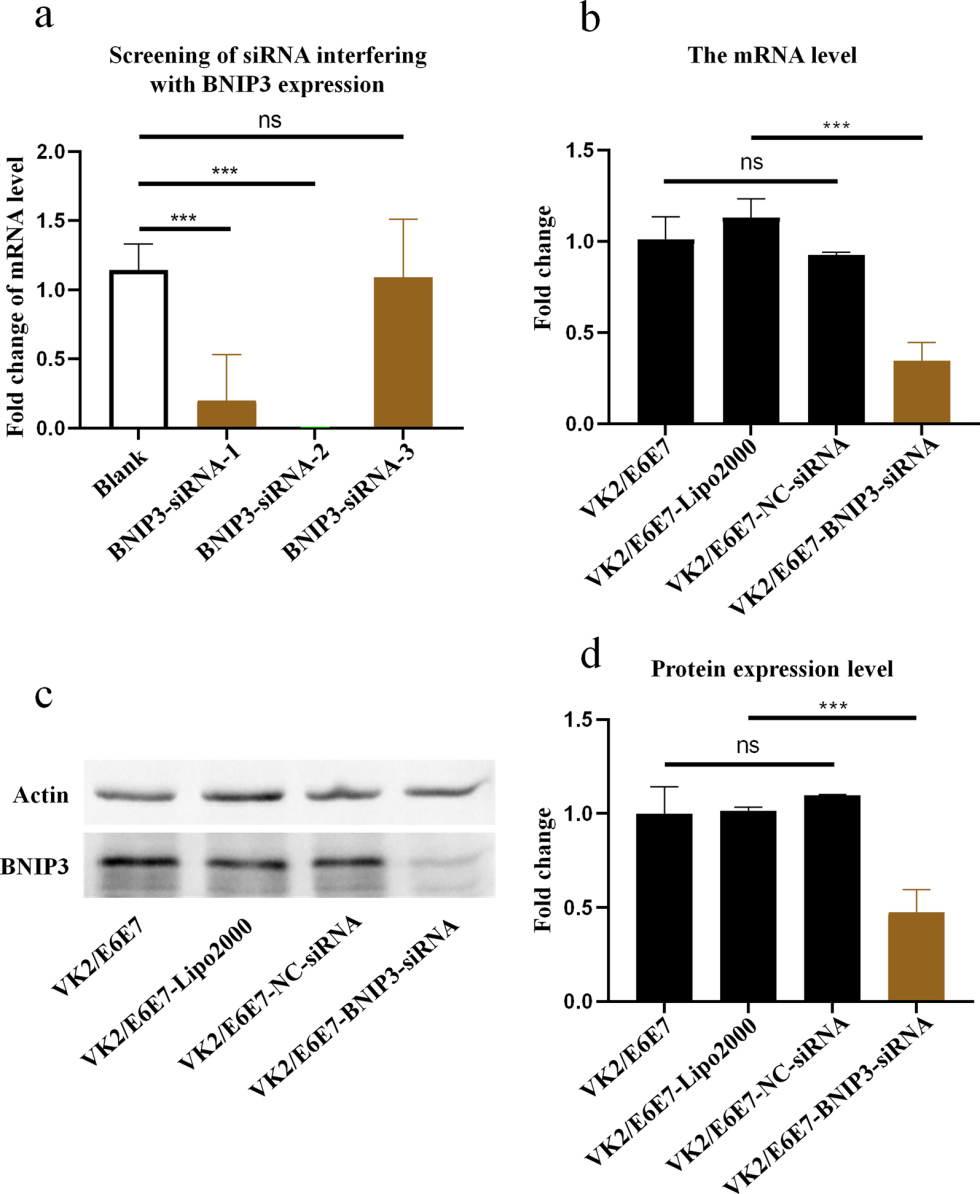
Expression of BNIP3 was knocked down by siRNA in VK2/E6E7 cells. **a** Screening the siRNA knocking down the mRNA level of BNIP3 by qPCR. **b** Changes of the mRNA level of BNIP3 in VK2/E6E7 cells after siRNA interference. **c** Changes in the protein level of BNIP3 in VK2/E6E7 cells after siRNA interference. Actin was used as internal reference protein. **d**. Histogram showing the relative expression levels of BNIP3 as measured in three independent experiments. *p* ≥ 0.05 and *p* < 0.001 represent statistical significances and was labeled as “ns” and “***”, respectively

The siRNA interfering with BNIP3 expression (Fig. 6a; Additional file 7: Figure S7) and the primers detecting the mRNA level of BNIP3 were screened by qPCR (Table 1; Additional file 8: Figure S8). After transfection of siRNA labeled with fluorescent dye to optimize the transfection conditions, we found that the optimal duration and siRNA concentration were 12 h and 100 nM, respectively, for transfecting siRNA into VK2/E6E7 cells (Additional file 9: Figure S9). Under the optimized conditions, siRNA interference was completed, and qPCR and western blot showed that the mRNA level and protein expression of BNIP3 were successfully knocked down by BNIP3-siRNA in VK2/E6E7 cells (Fig. 6b–d).

### Effect of the interaction between TvAP33 and BNIP3 on the infection of VK2/E6E7 cells by* T. vaginalis*

**Fig. 7:**
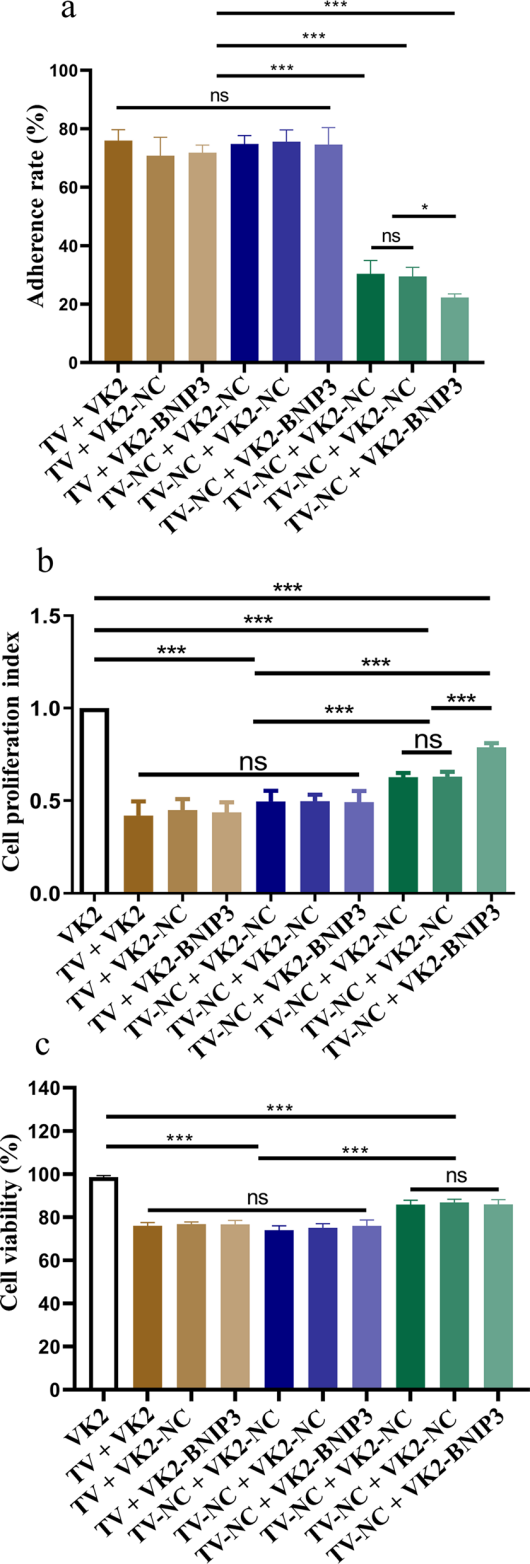
Effect of the interaction between TvAP33 and BNIP3 on the infection of VK2/E6E7 cells by* Trichomonas vaginalis*. **a** Analysis of the adherence rate of *T. vaginalis* on VK2/E6E7 cells, after the expression of TvAP33 and BNIP3 was knocked down in *T. vaginalis* and VK2/E6E7 cells, respectively. **b** Analysis of the proliferation of VK2/E6E7 cell by MTT assay, after the expression of TvAP33 and BNIP3 was knocked down in *T. vaginalis* and VK2/E6E7 cells, respectively. **c** Analysis of VK2/E6E7 cell viability, after the expression of TvAP33 and BNIP3 was knocked down in *T. vaginalis* and VK2/E6E7 cells, respectively. The results of adherence rate, cell proliferation index, and cell viability were presented as the mean ±  SD. *p* ≥0.05, *p* <0.05, *p* <0.01 and *p* <0.001 represent statistical significances and was labeled as “ns”, “*”, “**” and “***”, respectively.

VK2/E6E7 cells with BNIP3 knockdown were infected by *T. vaginalis* with TvAP33 knockdown, and the adhesion rate of trophozoites on VK2/E6E7 cells was calculated. The results of trophozoites adhering to VK2/E6E7 cells showed that the adhesion rates in the groups of *T. vaginalis* with normal expression of TvAP33 on the VK2/E6E7 cells with BNIP3 knockdown were not significantly changed, compared with those of the group of VK2/E6E7 cells infected with *T. vaginalis*. However, when the expression of TvAP33 was knocked down, the adhesion rates of trophozoites on VK2/E6E7 cells with BNIP3 knockdown were significantly lower than those of trophozoites on VK2/E6E7 cells with normal BNIP3 expression (Fig. 7a; Additional file 10: Figure S10), which suggested that: (i) the interaction between TvAP33 and BNIP3 played a role in the adhesion of *T. vaginalis* to host cells, but the interaction between TvAP33 and BNIP3 was not irreplaceable, and (ii) TvAP33 interacted with other molecules in VK2/E6E7 cells during the adhesion process.

Similarly, as shown in Fig. 7b, the proliferation of VK2/E6E7 cells with BNIP3 knockdown infected by *T. vaginalis* with normal expression of TvAP33 was not significantly changed, compared with the group of VK2/E6E7 cells infected with *T. vaginalis*, while the proliferation in the group of VK2/E6E7 cells with BNIP3 knockdown infected by *T. vaginalis* with TvAP33 knockdown was significantly higher than that in the groups of VK2/E6E7 cells with normal expression of BNIP3 infected by *T. vaginalis* with TvAP33 knockdown. These results indicate that the interaction between TvAP33 and BNIP3 could inhibit cell proliferation after host cells were infected with *T. vaginalis*, the interaction between these two molecules was not irreplaceable and other molecules in VK2/E6E7 cells were involved.

However, the expression level of BNIP3 in VK2/E6E7 cells did not significantly change cell viability during *T. vaginalis* infection (Fig. 7c; Additional file 11: Figure S11). Therefore, the interaction between TvAP33 and BNIP3 seemed to have no effect on host cell death induced by *T. vaginalis*.

## Discussion

Trichomoniasis, caused by *T. vaginalis*, is the most common nonviral sexually transmitted disease worldwide and presents a serious threat to human reproductive health. Oral metronidazole remains the recommended regimen for the treatment of trichomoniasis [27]. However, treatment failure does occur, mainly due to significant gastrointestinal adverse side effects of this medication, although these effects have been found to be temporary and disappear after the cessation of treatment [28]. The development of *T. vaginalis* resistance to nitroimidazoles is a growing problem, with up to 5% of clinical cases of vaginal trichomoniasis being associated with *T. vaginalis* strains with some level of resistance to nitroimidazoles [29–31]. Although many efforts to develop drugs to treat trichomoniasis have been made [32], so far there are no alternative drugs available in the clinic setting. Therefore, the development of new therapeutics with a novel mechanism of action is urgently needed. The attachment of *T. vaginalis*, an obligate extracellular pathogen, to the urogenital epithelium of both men and women allows the establishment and maintenance of an infection as well as nutrient acquisition from host cells [33, 34]. Despite adherence being crucial for this extracellular parasite to thrive within the host, relatively little is known about the mechanisms or key molecules involved in this attachment process. The results of some studies indicate that *T. vaginalis* adherence to the host epithelium is a multifaceted process in which a number of parasite factors play a role [18, 35–44].

The class of adhesion proteins is believed to be involved in the adhesion of trophozoites to host cells and this protein class has been extensively characterized in terms of cytoadherence; TvAP33 has been found to be a prominent functional protein in this process [45, 46]. TvAP33 gene expression has been reported to increase after the adherence of *T. vaginalis* to human vaginal epithelial cells [47]. A database search revealed that TvAP33 had significant identity to the α-SCS subunit of several different organisms and virtually 100% identity to the reported *T. vaginalis* subunit [19]. Mundodi et al. confirmed the role of the TvAP33 protein as an adhesin in *T. vaginalis* [20]. Moreover, Liang et al. indicated that the expressed fusion protein TvAP33 showed satisfactory antigenicity and immunogenicity [48]. TvAP33 could be used as a vaccine candidate antigen for anti-*T. vaginalis* infection [24]. However, some reports indicated that the evidence supporting a role of adhesion proteins in adhesion of *T. vaginalis* cells was indirect or controversial [25, 33] and that these adhesin proteins lacked several characteristics of ‘true’ adhesion molecules [25]. With the exception of TvAP23, other adhesion proteins were indicated as abundant metabolic enzymes primarily involved in carbohydrate metabolism and found in the hydrogenosome, a mitochondrion-like organelle found in *T. vaginalis* [49]. The main reason why there was such a dispute is that few studies were able to directly prove that the lack of adhesion proteins affected the adhesion of *T. vaginalis*. In the current study, we found that the number of *T. vaginalis* trophozoites adhering to VK2/E6E7 cells decreased significantly after the expression of TvAP33 was knocked down, which confirmed that TvAP33 was indeed involved in the process of *T. vaginalis* adhering to host cells. This result is consistent with that reported by Mundodi et al. [20]. Our results also showed that by knocking down the expression of TvAP33, trophozoites significantly decreased the inhibition of VK2/E6E7 cell proliferation and reduced VK2/E6E7 cell apoptosis and death. These results support the notion that TvAP33 mediates the adhesion of *T. vaginalis* to host cells, thus affecting the activity of host cells. However, whether TvAP33 directly damages host cells as a virulence factor needs further study.

In addition, in this study, the animal challenge assay showed that the longevity of mice infected with trophozoites blocked by TvAP33 polyclonal antibody were significantly prolonged, which was consistent with the result of our previous study that TvAP33 could act as a vaccine candidate antigen against *T. vaginalis* infection [24]. All of these results suggested that the adhesion mediated by TvAP33 positively affected the pathogenicity of *T. vaginalis*. We also found that TvAP65 could be used as a vaccine candidate antigen to induce cell-mediated and humoral immunity against *T. vaginalis* infection [26]. However, whether TvAP65 affects the pathogenicity of *T. vaginalis* through an adhesion role similar to TvAP33 needs further research.

In the present study, the TvAP33 protein was characterized further to identify domains interacting with host surface sites, and the results showed that the – and C-termini of TvAP33 could bind to host cells [21]. TvAP33 and TvAP51 were characterized as α- and β-SCS, which were biotinylated in the cytosol and imported exclusively into the hydrogenosomes. Neither α- nor β-SCS was biotinylated in the endoplasmic reticulum and delivered to the cell surface via the secretory pathway [22]. However, it is still unclear which molecular interactions between TvAP33 and host cells mediate adhesion and pathogenesis. In this study, we found that the rTvAP33 protein could bind to VK2/E6E7 cells, and we identified 18 proteins interacting with TvAP33 in VK2/E6E7 cells. These identified molecules were mainly involved in energy metabolism, signal transduction, cell proliferation and differentiation, cell cycle progression and wound repair and cell apoptosis.

Heme oxygenase catalyzes the degradation of heme to carbon monoxide (CO), iron and biliverdin [50]. The action of heme oxygenase facilitates the synthesis of iron-binding proteins and, hence, cellular iron storage and transport [51]. *Trichomonas vaginalis* has been reported to present a high iron dependency to regulate its growth, metabolism, and virulence properties [52]. The interaction between TvAP33 and heme oxygenase protein may be a manner for *T. vaginalis* to obtain iron from host cells. Moreover, many stimuli involved in the pathogenesis of renal injury, such as heme, nitric oxide, cytokines, ischemia, lipopolysaccharides, irradiation and nephrotoxins, induce overexpression of heme oxygenase, and the induction of heme oxygenase occurs as an adaptive and beneficial response to these stimuli, as demonstrated by different studies, due to its vasorelaxant, anti-inflammatory and antiapoptotic actions [53]. However, whether the interaction between TvAP33 and heme oxygenase is involved in the parasitism and pathogenesis of *T. vaginalis* in host cells needs further study.

BNIP3 is a mitochondrial BH3-only member of the Bcl-2 protein family, and the protein is activated by hypoxia; it mainly functions as a cell death regulator under hypoxic conditions [54]. BNIP3 affects different cell death pathways, including apoptosis, necrosis-like cell death, autophagy and its special form, mitophagy. These processes are also involved in epithelial-mesenchymal transition (EMT) and metastasis [55]. In addition to participating in cell death and metastasis-related processes, BNIP3 can control different metabolic pathways, such as lipid metabolism [56], glycolysis [57] and mitochondrial bioenergetics [58]. In the current study, we screened the BNIP3 protein interacting with the TvAP33 protein in vaginal epithelial cells by yeast two-hybrid assays, and this interaction was verified by co-immunoprecipitation and immunofluorescence. In addition, immunofluorescence results showed that TvAP33 and BNIP3 were colocated in the cell membrane and cytoplasm. Nievas et al. indicated that *T. vaginalis* released microvesicle-like structures (MVs) [59]. These authors considered MVs to be universal transport vehicles for intercellular communication as they could incorporate peptides, proteins, lipids, microRNA and mRNA, all of which could be transferred to target cells through receptor-ligand interactions, fusion with the cell membrane and delivery of a functional cargo to the cytoplasm of the target cell [59]. Therefore, TvAP33 might be transported into the host cell through MVs and interact with BNIP3.

Since BNIP3, as a multifunctional protein, can interact with TvAP33, we investigated whether BNIP3 played a role in mediating the adhesion of *T. vaginalis* to host cells and influencing the activity of host cells through an interaction with TvAP33. To address this issue, we knocked down the expression of BNIP3 in VK2/E6E7 cells and then analyzed the adhesion rate of trophozoites and the proliferation and viability of VK2/E6E7 cells after *T. vaginalis* infection of VK2/E6E7 cells with low BNIP3 expression. We found that the adhesion rates of *T. vaginalis* were not significantly changed after only knocking down BNIP3 in VK2/E6E7 cells, but when the expression of TvAP33 in *T. vaginalis* was knocked down, the adhesion rates of trophozoites on VK2/E6E7 cells with BNIP3 knockdown were significantly lower than those of trophozoites on VK2/E6E7 cells with normal BNIP3 expression; these results indicate that the interaction between TvAP33 and BNIP3 could positively affect the adhesion of *T. vaginalis* to host cells, but that the interaction was not irreplaceable and TvAP33 interacted with other molecules in VK2/E6E7 cells during the adhesion process. Recent studies have shown that BNIP3 can regulate many fundamental cellular functions, such as mitochondrial quality control, proliferation, differentiation and maturation [60]; thus, BNIP3 might act as a potential link between the death and survival of the cell [61]. On possibility is that TvAP33 might promote adhesion of *T. vaginalis* to host cells by interacting with BNIP3 to reduce cell activity. In the present study, we found that compared with that of the group of VK2/E6E7 cells infected with *T. vaginalis*, the proliferation of VK2/E6E7 cells with BNIP3 knockdown infected by *T. vaginalis* with normal expression of TvAP33 was not significantly changed, while the proliferation in the group of VK2/E6E7 cells with BNIP3 knockdown infected by *T. vaginalis* with TvAP33 knockdown was significantly higher than that in the groups of VK2/E6E7 cells with normal expression of BNIP3 infected by *T. vaginalis* with TvAP33 knockdown. Similar to the role of BNIP3 in the adhesion of *T. vaginalis* to host cells, the interaction between TvAP33 and BNIP3 could inhibit host cell proliferation after host cells were infected with *T. vaginalis*, but the interaction between these two molecules was not irreplaceable, and other molecules in VK2/E6E7 cells were involved. BNIP3 could induce classical apoptosis through cytochrome c and caspase activation. However, the expression level of BNIP3 in VK2/E6E7 cells did not significantly change cell viability during *T. vaginalis* infection, and the results implied that TvAP33 did not induce host cell apoptosis and death through the BNIP3 pathway in *T. vaginalis* infection. Therefore, the molecular mechanism of TvAP33 reducing host cell activity in *T. vaginalis* infection needs further study.

## Conclusions

As an extracellular parasitic protozoan, *T. vaginalis* adheres to the epithelial lining of the host's urogenital tract to survive. The adherence to the surface of the host cell via TvAP33 is the precondition for parasitism and pathogenicity of this parasite. However, the molecular mechanism of adhesion and pathogenicity of *T. vaginalis* to host cells mediated by TvAP33 is unclear. In the present study, we knocked down the expression of TvAP33 in trophozoites by siRNA and found that TvAP33 played an important role in adhesion to host cells, inhibition of host cell proliferation and induction of host cell apoptosis and death during the process of *T. vaginalis* infection. The results of animal challenge experiments showed that the pathogenicity of trophozoites was decreased by passive immunization with TvAP33 antiserum or blocking of the TvAP33 protein. Immunofluorescence analysis showed that TvAP33 could bind to VK2/E6E7 cells, and 13 protein molecules interacting with TvAP33 were screened from the cDNA library of VK2/E6E7 cells by a yeast two-hybrid system. Moreover, the interaction between TvAP33 and BNIP3 was further confirmed by co-immunoprecipitation and immunofluorescence. Through *T. vaginalis* with low expression of TvAP33 infecting VK2/E6E7 cells with low expression of BNIP3, we revealed that the interaction between TvAP33 and BNIP3 could promote the adhesion of *T. vaginalis* to host cell and inhibit host cells proliferation, but the interaction was not irreplaceable, and other molecules in host cells were involved during the adhesion process. However, the interaction between these two molecules seemed to have no effect on host cell death induced by *T. vaginalis*.

 In conclusion, our study demonstrated that the interaction between TvAP33 and BNIP3 mediated the adhesion and pathogenicity of *T. vaginalis* to host cells, although the role of the interaction of TvAP33 and BNIP3 in the pathogenesis of *T. vaginalis* infecting host cells was not unique. These results provide a basis for searching the drug targets of *T. vaginalis* and put forward new ideas for the prevention and treatment of trichomoniasis.

## Supplementary Information

**Additional file 1: Figure S1.** Amplification efficiency and specificity of TvAP33 and TvActin primers used for qPCR in this research. TvActin was used as internal reference gene in detecting the mRNA level of TvAP33 by qPCR.** A** Standard curve of primer amplification efficiency for qPCR. A1, TvAP33; A2, TvActin.** B** Melt curve of primers used for qPCR. B1, TvAP33; B2, TvActin.

**Additional file 2: Figure S2.** The 3 alternative siRNAs. The mRNA level of TvAP33 after 3 alternative siRNAs interfered with *T. vaginalis* trophozoites. Asterisks indicate statistically significant difference at *P < 0.05, **P < 0.01; ns indicates absence of significance (*P* ≥ 0.05). TvAP33-siRNA1 was used to subsequently knock down the expression of TvAP33.

**Additional file 3: Figure S3.** Optimization of the transfection conditions. **A** The siRNA concentration was optimized by transfecting siRNA labeled with fluorescent dye to *T. vaginalis* trophozoites. **B** The mRNA level of TvAP33 was analyzed by qPCR, after siRNA transfection of *T. vaginalis* trophozoites for 24 h. **C** The mRNA level of TvAP33 were analyzed by qPCR, after siRNA transfection of* T. vaginalis* trophozoites for 36 h. Asterisks indicate statistically significant difference at *P < 0.05, **P < 0.01; ns indicates absence of significance (*P* ≥ 0.05).

**Additional file 4: Figure S4.** Quality inspection of the cDNA Library of VK2/E6E7 cells. **A** Agarose gel electrophoresis of total RNA in VK2/E6E7 cells. Lanes:* M* DNA molecular weight marker DL 5000 (ordinate values in bp),* 1* total RNA of VK2/E6E7 cells. **B** Agarose gel electrophoresis of mRNA in VK2/E6E7 cells. Lanes:* M *DNA molecular weight marker DL 2000 (ordinate values in bp),* 1* mRNA of VK2/E6E7 cells. **C** Agarose gel electrophoresis of the double-stranded cDNA. Lanes:* M* DNA molecular weight marker DL 2000 (ordinate values in bp),* 1* double-stranded cDNA. **D** Analysis of the recombination efficiency and the inserted fragment in primary library. Lanes:* M* DNA molecular weight marker DL 2000 (ordinate values in bp),* 1-24* PCR analysis of 24 bacterial colonies. **E** Analysis of the recombination efficiency and the inserted fragment in secondary library. Lanes:* M* DNA molecular weight marker DL 2000 (ordinate values in bp),* 1-24* PCR analysis of 24 bacterial colonies. **F** Identification of primary recombinants. **G** Identification of secondary recombinants.

**Additional file 5: Figure S5.** Examination of the functionality and self-activation of yeast two hybrid system. **A** Verification of the functionality of yeast two hybrid system. The plasmids of PTSU2-APP and pNubG-Fe65 were used as positive bait vector and positive capture vector, respectively. The pPR3N plasmid was used to construct the cDNA library of VK2/E6E7 cells. **B** Examination of the self-activation of yeast two hybrid system. The plasmid of pOst1-NubI was used as positive bait control. The vector of pDHB1-TvAP33 was used to fish the protein molecules interacting with TvAP33 from cDNA library of VK2/E6E7 cells.

**Additional file 6: Figure S6.** The screened molecules were co-transformed into yeast cells with bait TvAP33 to verify the interaction, respectively.

**Additional file 7: Figure S7.** The 3 alternative siRNAs.

**Additional file 8: Figure S8.** Amplification efficiency and specificity of BNIP3 and actin primers used for qPCR in this research. Actin was used as internal reference gene in detecting the mRNA level of BNIP3 by qPCR. **A** Standard curve of primer amplification efficiency for qPCR. A1, BNIP3; A2, actin. **B** Melt curve of primers used for qPCR. B1, BNIP3; B2, actin.

**Additional file 9: Figure S9.** Optimization of the transfection conditions by transfecting siRNA labeled with fluorescent dye to VK2/E6E7. **A** Screening the optimal duration for transfecting siRNA into VK2/E6E7. A1, 6 h; A2, 12 h; A3, 24 h. **B** Screening the optimal siRNA concentration for transfecting siRNA into VK2/E6E7. B1, 50 nM; B2, 100 nM; B2, 150 nM.

**Additional file 10: Figure S10.** Observation of *T. vaginalis* adherence to VK2/E6E7 cells under the fluorescence microscope (40×). The trophozoites were stained with red fluorescence by CytoTraceTM Orange.

**Additional file 11: Figure S11.** Observation of VK2/E6E7 cells viability under the microscope (40×). These dead cells were stained with blue by trypan blue. **A** VK2/E6E7 cells, **B** *T. vaginalis + *VK2/E6E7 cells, **C** *T. vaginalis + *VK2/E6E7-NC-siRNA cells, **D** *T. vaginalis + *VK2/E6E7-BNIP3-siRNA cells, **E** *T. vaginalis-*NC-siRNA* + *VK2/E6E7 cells, **F** *T. vaginalis-*NC-siRNA* + *VK2/E6E7-NC-siRNA cells, **G** *T. vaginalis-*NC-siRNA* + *VK2/E6E7-BNIP3-siRNA cells, **H** *T. vaginalis-*TvAP33-siRNA* + *VK2/E6E7 cells, **I** *T. vaginalis-*TvAP33-siRNA* + *VK2/E6E7-NC-siRNA cells, **J** *T. vaginalis-*TvAP33-siRNA* + *VK2/E6E7-BNIP3-siRNA cells.

## Data Availability

All data generated or analyzed during this study are included in this article and additional information files.
