# Supplementary material for: The interaction between adhesion protein 33 (TvAP33) and BNIP3 mediates the adhesion and pathogenicity of Trichomonas vaginalis to host cells
Source: Parasit Vectors. 2023 Jun 21;16:210. doi: 10.1186/s13071-023-05798-x (PMC10286359; doi:10.1186/s13071-023-05798-x)
Supplement: Supplementary file 1 — Additional file 1: Figure S1. Amplification efficiency and specificity of TvAP33 and TvActin primers used for qPCR in this research. TvActin was used as internal reference gene in detecting the mRNA level of TvAP33 by qPCR. A Standard curve of primer amplification efficiency for qPCR. A1, TvAP33; A2, TvActin. B Melt curve of primers used for qPCR. B1, TvAP33; B2, TvActin. [file 13071_2023_5798_MOESM1_ESM.docx]

Additional 1


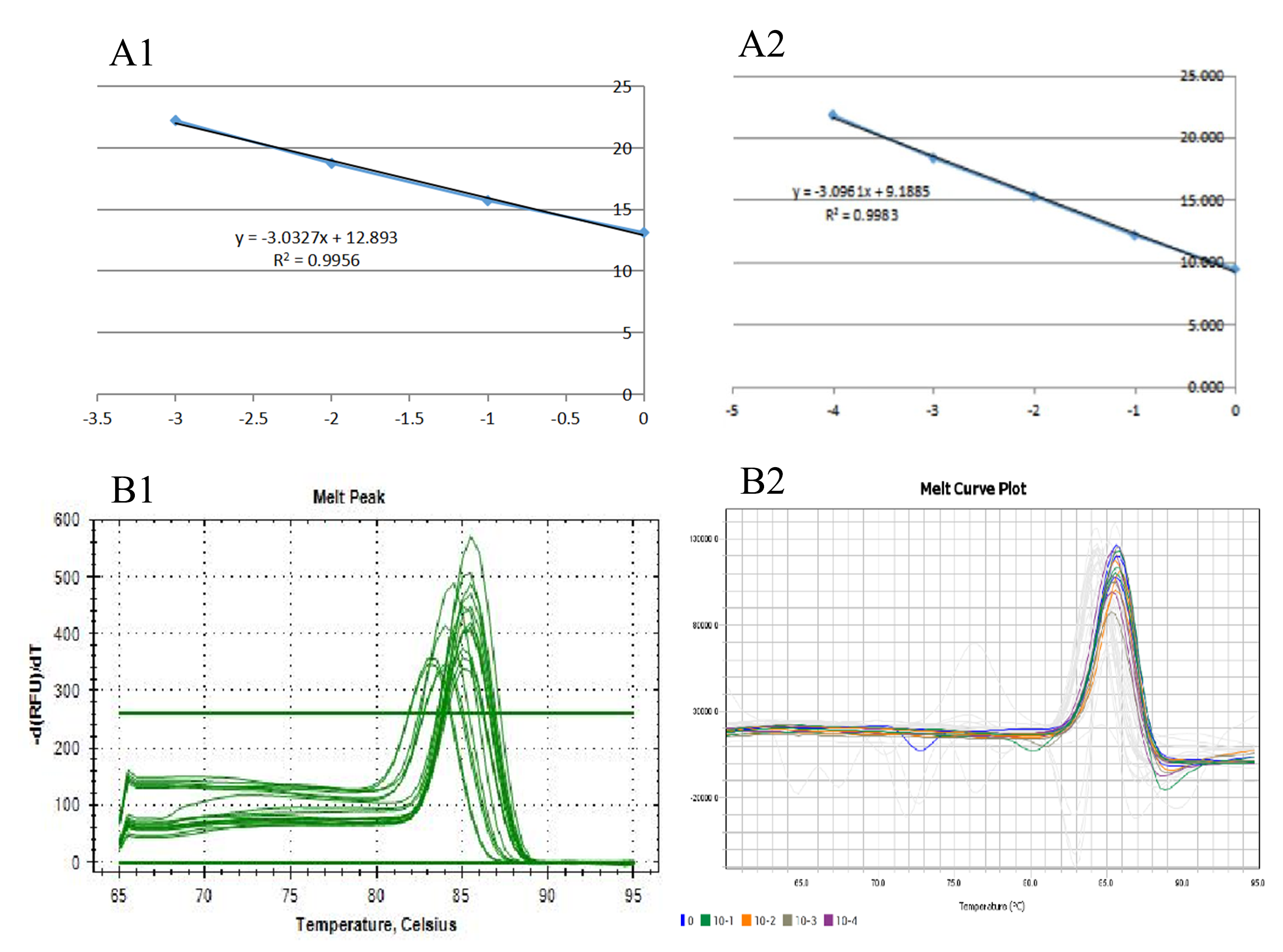


Additional 1 Legend

Amplification efficiency and specificity of TvAP33 and TvActin primers used for qPCR in this research. TvActin was used as internal reference gene in detecting the mRNA level of TvAP33 by qPCR. A: Standard curve of primer amplification efficiency for qPCR. A1: TvAP33. A2: TvActin. B: Melt curve of primers used for qPCR. B1: TvAP33. B2: TvActin.
