# Supplementary material for: The interaction between adhesion protein 33 (TvAP33) and BNIP3 mediates the adhesion and pathogenicity of Trichomonas vaginalis to host cells
Source: Parasit Vectors. 2023 Jun 21;16:210. doi: 10.1186/s13071-023-05798-x (PMC10286359; doi:10.1186/s13071-023-05798-x)
Supplement: Supplementary file 2 — Additional file 2: Figure S2. The 3 alternative siRNAs. The mRNA level of TvAP33 after 3 alternative siRNAs interfered with T. vaginalis trophozoites. Asterisks indicate statistically significant difference at *P < 0.05, **P < 0.01; ns indicates absence of significance (P ≥ 0.05). TvAP33-siRNA1 was used to subsequently knock down the expression of TvAP33. [file 13071_2023_5798_MOESM2_ESM.docx]

Additional 2

Table The 3 alternative siRNAs

| Name | siRNA Sequence (5'→3') |
| --- | --- |
| **TvAP33-siRNA1 sense** | **GUCUCCCAAUCUUCAAGAACATT** |
| **TvAP33-siRNA1 antisense** | **UGUUCUUGAAGAUUGGGAGACTT** |
| TvAP33-siRNA2 sense | GGAUCGCUAAGACAAAGCUUATT |
| TvAP33-siRNA2 antisense | UAAGCUUUGUCUUAGCGAUCCTT |
| TvAP33-siRNA3 sense | CAGGUAACAUGGGCAAGUUCATT |
| TvAP33-siRNA3 antisense | UGAACUUGCCCAUGUUACCUGTT |

Figure

Figure Legend

The mRNA level of TvAP33 after three alternative siRNAs interfered with *T. vaginalis* trophozoites. p≥0.05, p <0.05, p <0.01 and p <0.001 represent statistical significances and was labeled as “ns”, “*”, “**” and “***”, respectively. TvAP33-siRNA1 was used to subsequently knock down the expression of TvAP33.
