# Supplementary material for: The interaction between adhesion protein 33 (TvAP33) and BNIP3 mediates the adhesion and pathogenicity of Trichomonas vaginalis to host cells
Source: Parasit Vectors. 2023 Jun 21;16:210. doi: 10.1186/s13071-023-05798-x (PMC10286359; doi:10.1186/s13071-023-05798-x)
Supplement: Supplementary file 3 — Additional file 3: Figure S3. Optimization of the transfection conditions. A The siRNA concentration was optimized by transfecting siRNA labeled with fluorescent dye to T. vaginalis trophozoites. B The mRNA level of TvAP33 was analyzed by qPCR, after siRNA transfection of T. vaginalis trophozoites for 24 h. C The mRNA level of TvAP33 were analyzed by qPCR, after siRNA transfection of T. vaginalis trophozoites for 36 h. Asterisks indicate statistically significant difference at *P < 0.05, **P < 0.01; ns indicates absence of significance (P ≥ 0.05). [file 13071_2023_5798_MOESM3_ESM.docx]

Additional 3

Figure

A


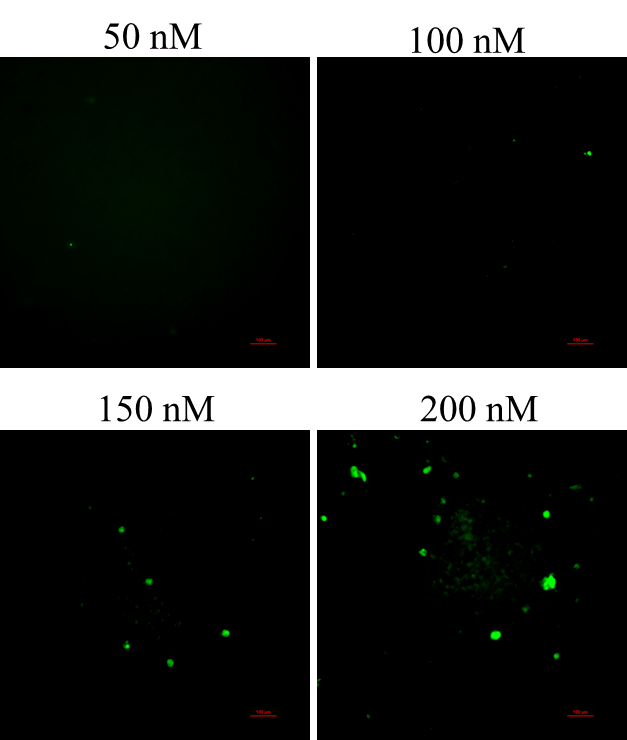


B


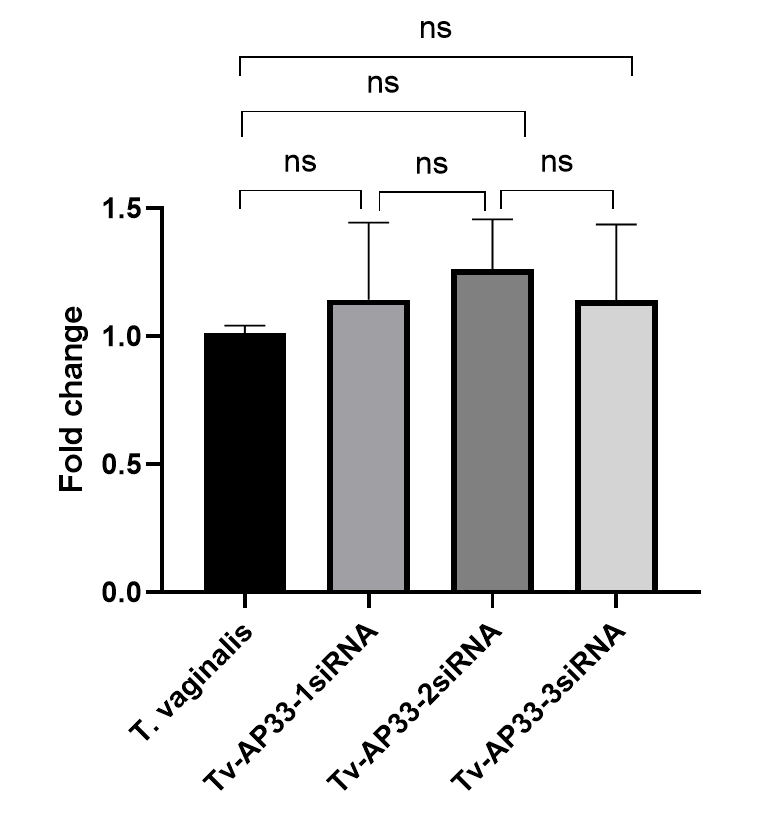


C


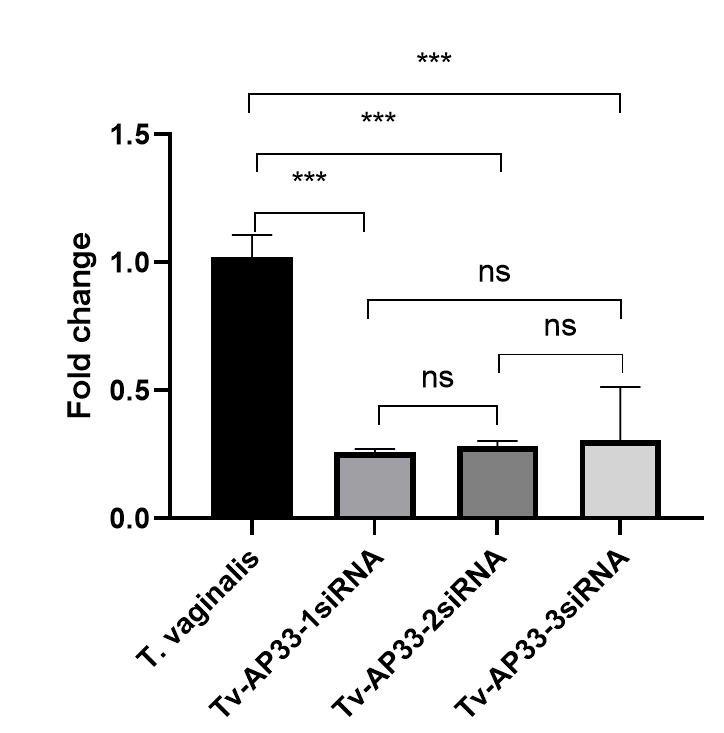


Figure Legend

Optimize the transfection conditions. A: The siRNA concentration was optimized by transfecting siRNA labeled with fluorescent dye to *T. vaginalis* trophozoites. B: The mRNA level of TvAP33 were analysed by qPCR, after siRNA transfection of *T. vaginalis* trophozoites for 24 hours. C: The mRNA level of TvAP33 were analysed by qPCR, after siRNA transfection of *T. vaginalis* trophozoites for 36 hours. p≥0.05, p <0.05, p <0.01 and p <0.001 represent statistical significances and was labeled as “ns”, “*”, “**” and “***”, respectively.
