# Supplementary material for: The interaction between adhesion protein 33 (TvAP33) and BNIP3 mediates the adhesion and pathogenicity of Trichomonas vaginalis to host cells
Source: Parasit Vectors. 2023 Jun 21;16:210. doi: 10.1186/s13071-023-05798-x (PMC10286359; doi:10.1186/s13071-023-05798-x)
Supplement: Supplementary file 4 — Additional file 4: Figure S4. Quality inspection of the cDNA Library of VK2/E6E7 cells. A Agarose gel electrophoresis of total RNA in VK2/E6E7 cells. Lanes: M DNA molecular weight marker DL 5000 (ordinate values in bp), 1 total RNA of VK2/E6E7 cells. B Agarose gel electrophoresis of mRNA in VK2/E6E7 cells. Lanes: M DNA molecular weight marker DL 2000 (ordinate values in bp), 1 mRNA of VK2/E6E7 cells. C Agarose gel electrophoresis of the double-stranded cDNA. Lanes: M DNA molecular weight marker DL 2000 (ordinate values in bp), 1 double-stranded cDNA. D Analysis of the recombination efficiency and the inserted fragment in primary library. Lanes: M DNA molecular weight marker DL 2000 (ordinate values in bp), 1-24 PCR analysis of 24 bacterial colonies. E Analysis of the recombination efficiency and the inserted fragment in secondary library. Lanes: M DNA molecular weight marker DL 2000 (ordinate values in bp), 1-24 PCR analysis of 24 bacterial colonies. F Identification of primary recombinants. G Identification of secondary recombinants. [file 13071_2023_5798_MOESM4_ESM.docx]

Additional 4

Figure


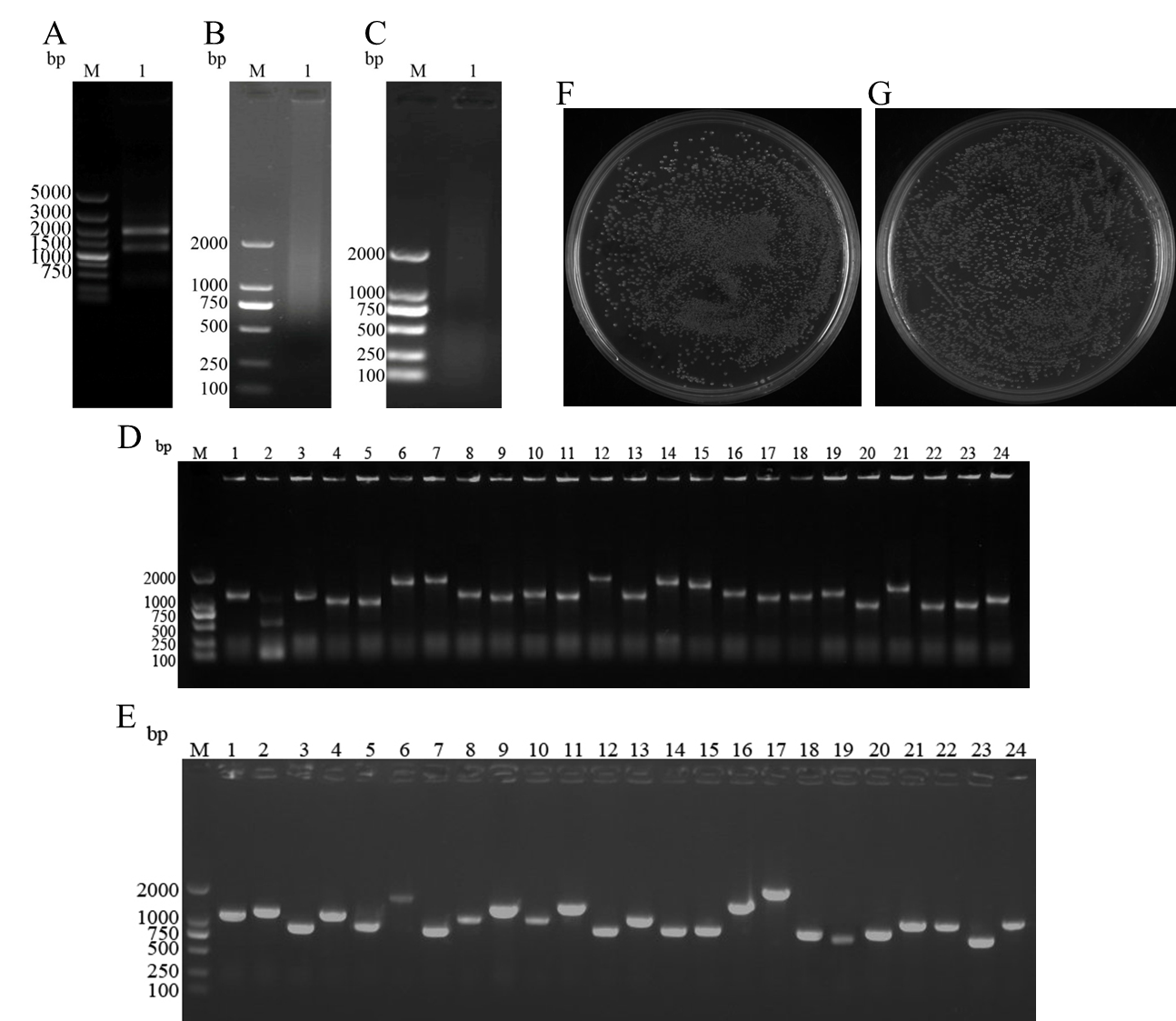


Figure Legend

Quality inspection of the cDNA Library of VK2/E6E7 cells. A: Agarose gel electrophoresis of total RNA in VK2/E6E7 cells. (Lane M) DNA molecular weight marker DL 5000 (ordinate values in bp); (Lane 1) The total RNA of VK2/E6E7 cells. B: Agarose gel electrophoresis of mRNA in VK2/E6E7 cells. (Lane M) DNA molecular weight marker DL 2000 (ordinate values in bp); (Lane 1) The mRNA of VK2/E6E7 cells. C: Agarose gel electrophoresis of the double-stranded cDNA. (Lane M) DNA molecular weight marker DL 2000 (ordinate values in bp); (Lane 1) The double-stranded cDNA. D: Analysis of the recombination efficiency and the inserted fragment in primary library. (Lane M) DNA molecular weight marker DL 2000 (ordinate values in bp); (Lane 1-24) PCR analysis of 24 bacterial colonies; E: Analysis of the recombination efficiency and the inserted fragment in secondary library. (Lane M) DNA molecular weight marker DL 2000 (ordinate values in bp); (Lane 1-24) PCR analysis of 24 bacterial colonies; F: Identification of primary recombinants. G: Identification of secondary recombinants.
