# Supplementary material for: The interaction between adhesion protein 33 (TvAP33) and BNIP3 mediates the adhesion and pathogenicity of Trichomonas vaginalis to host cells
Source: Parasit Vectors. 2023 Jun 21;16:210. doi: 10.1186/s13071-023-05798-x (PMC10286359; doi:10.1186/s13071-023-05798-x)
Supplement: Supplementary file 5 — Additional file 5: Figure S5. Examination of the functionality and self-activation of yeast two hybrid system. A Verification of the functionality of yeast two hybrid system. The plasmids of PTSU2-APP and pNubG-Fe65 were used as positive bait vector and positive capture vector, respectively. The pPR3N plasmid was used to construct the cDNA library of VK2/E6E7 cells. B Examination of the self-activation of yeast two hybrid system. The plasmid of pOst1-NubI was used as positive bait control. The vector of pDHB1-TvAP33 was used to fish the protein molecules interacting with TvAP33 from cDNA library of VK2/E6E7 cells. [file 13071_2023_5798_MOESM5_ESM.docx]

Additional 5

Figure


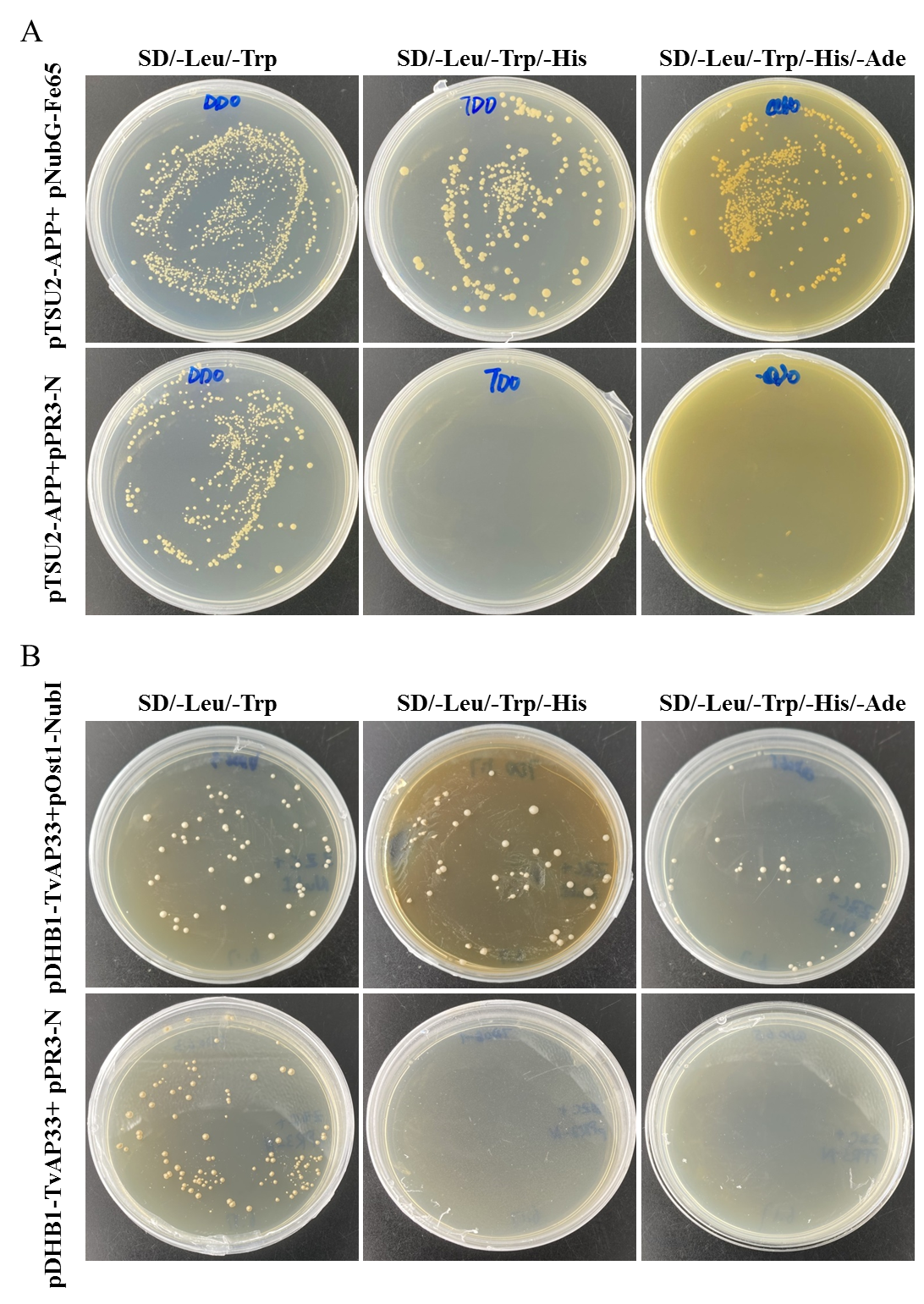


Figure Legend

The examination of the functionality and self-activation of yeast two hybrid system. A: Verification of the functionality of yeast two hybrid system. The plasmids of PTSU2-APP and pNubG-Fe65 were used as positive bait vector and positive capture vector, respectively. The pPR3N plasmid was used to construct the cDNA library of VK2/E6E7 cells. B: Examination of the self-activation of yeast two hybrid system. The plasmid of pOst1-NubI was used as positive bait control. The vector of pDHB1-TvAP33 was used to fish the protein molecules interacting with TvAP33 from cDNA library of VK2/E6E7 cells.
