# Supplementary material for: The interaction between adhesion protein 33 (TvAP33) and BNIP3 mediates the adhesion and pathogenicity of Trichomonas vaginalis to host cells
Source: Parasit Vectors. 2023 Jun 21;16:210. doi: 10.1186/s13071-023-05798-x (PMC10286359; doi:10.1186/s13071-023-05798-x)
Supplement: Supplementary file 6 — Additional file 6: Figure S6. The screened molecules were co-transformed into yeast cells with bait TvAP33 to verify the interaction, respectively. [file 13071_2023_5798_MOESM6_ESM.docx]

Additional 6

Figure

**SD/-Leu/-Trp/-His/-Ade**

**SD/-Leu/-Trp**

**SD/-Leu/-Trp**

**SD/-Leu/-Trp/-His/-Ade**


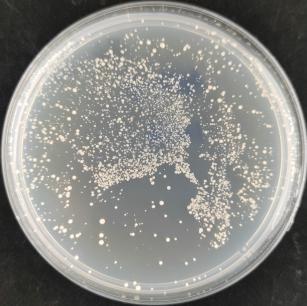

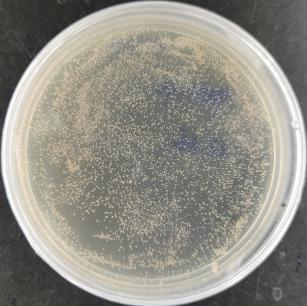

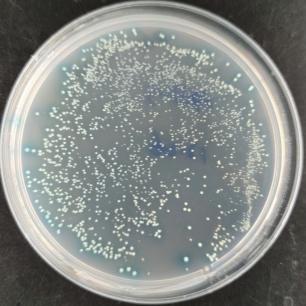

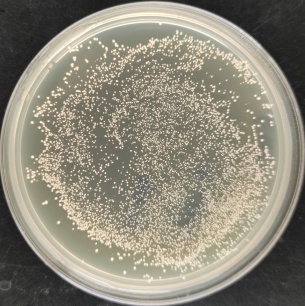


**AP33-4**

**AP33-3**


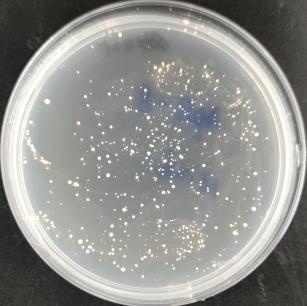



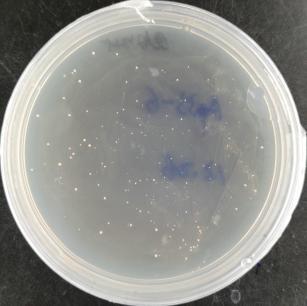

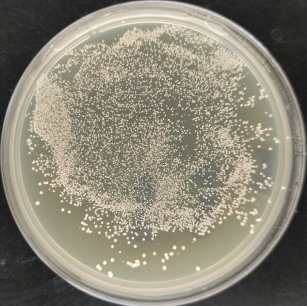


**AP33-8**

**AP33-6**


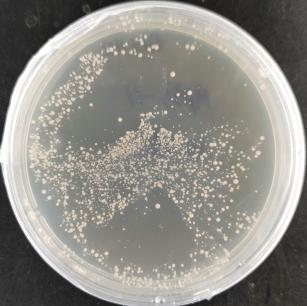

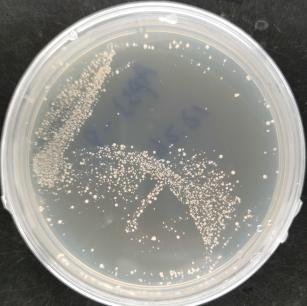

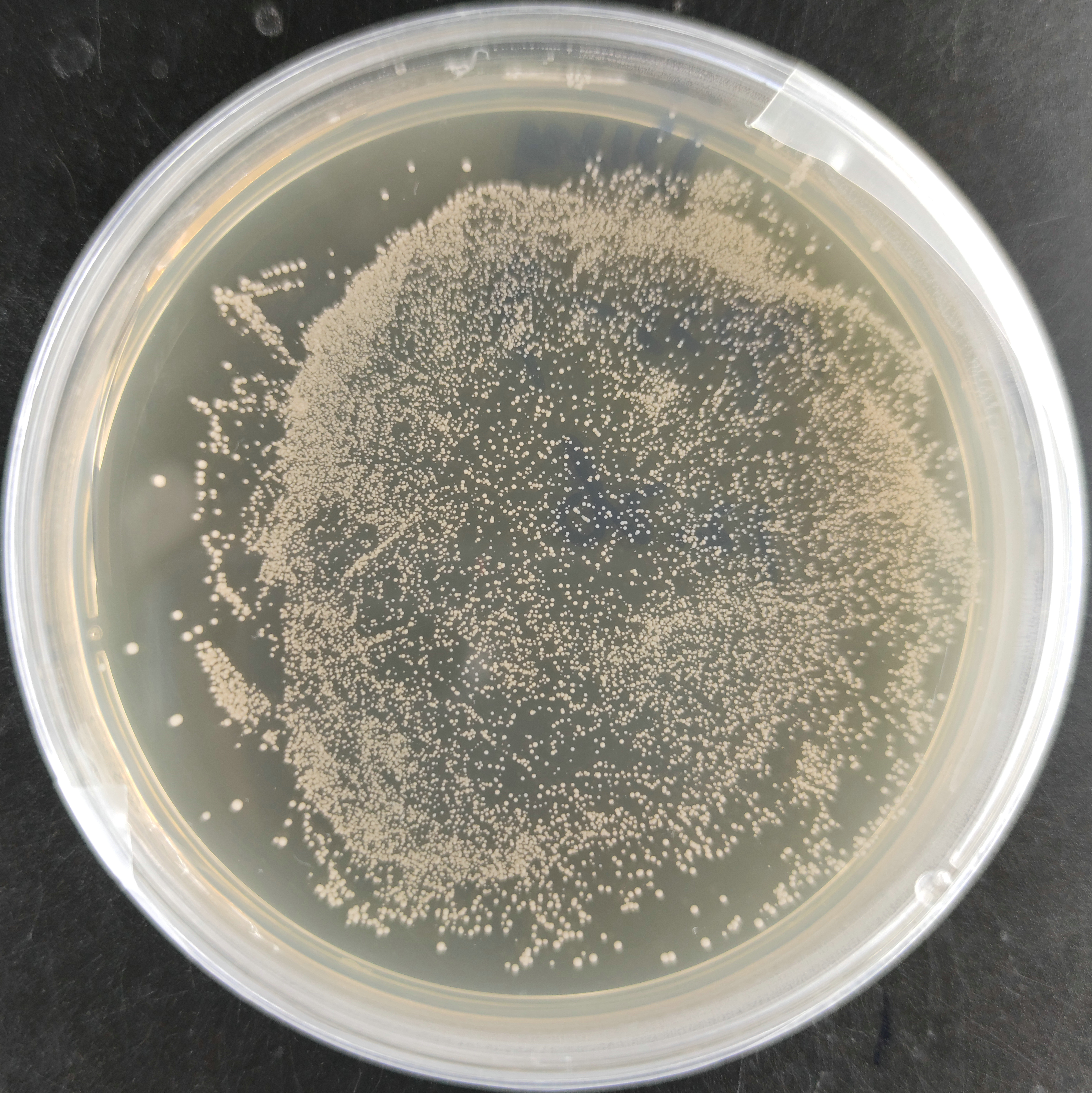

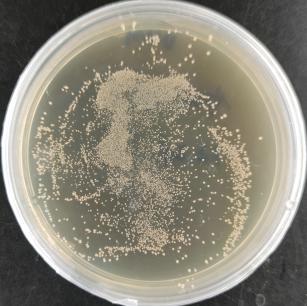


**AP33-9**

**AP33-11**


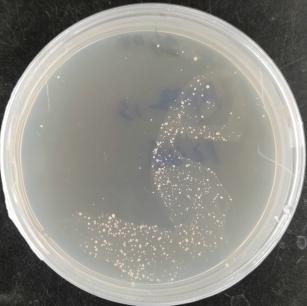

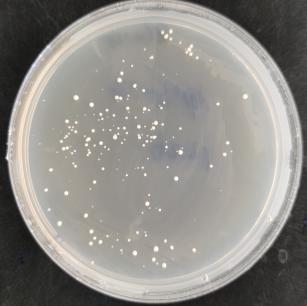

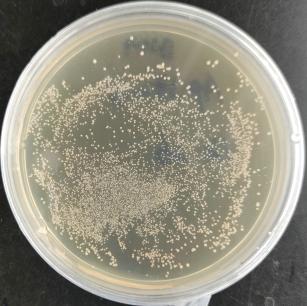

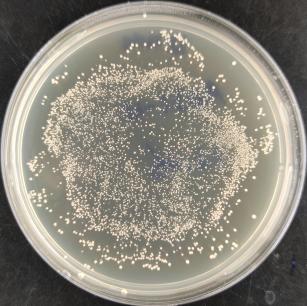


**AP33-12**

**AP33-13**


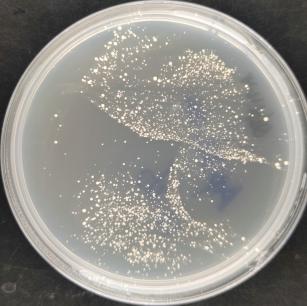

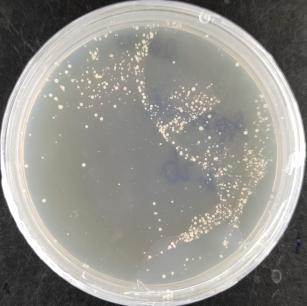

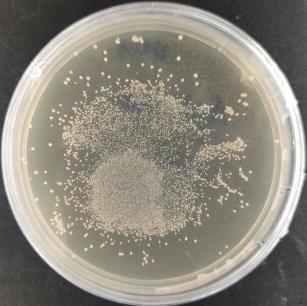

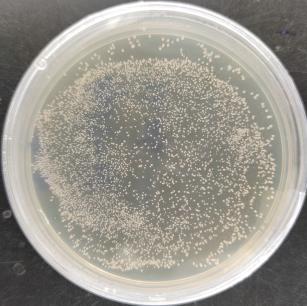


**AP33-16**

**AP33-22**


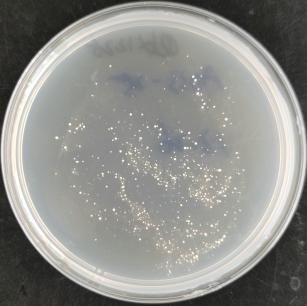

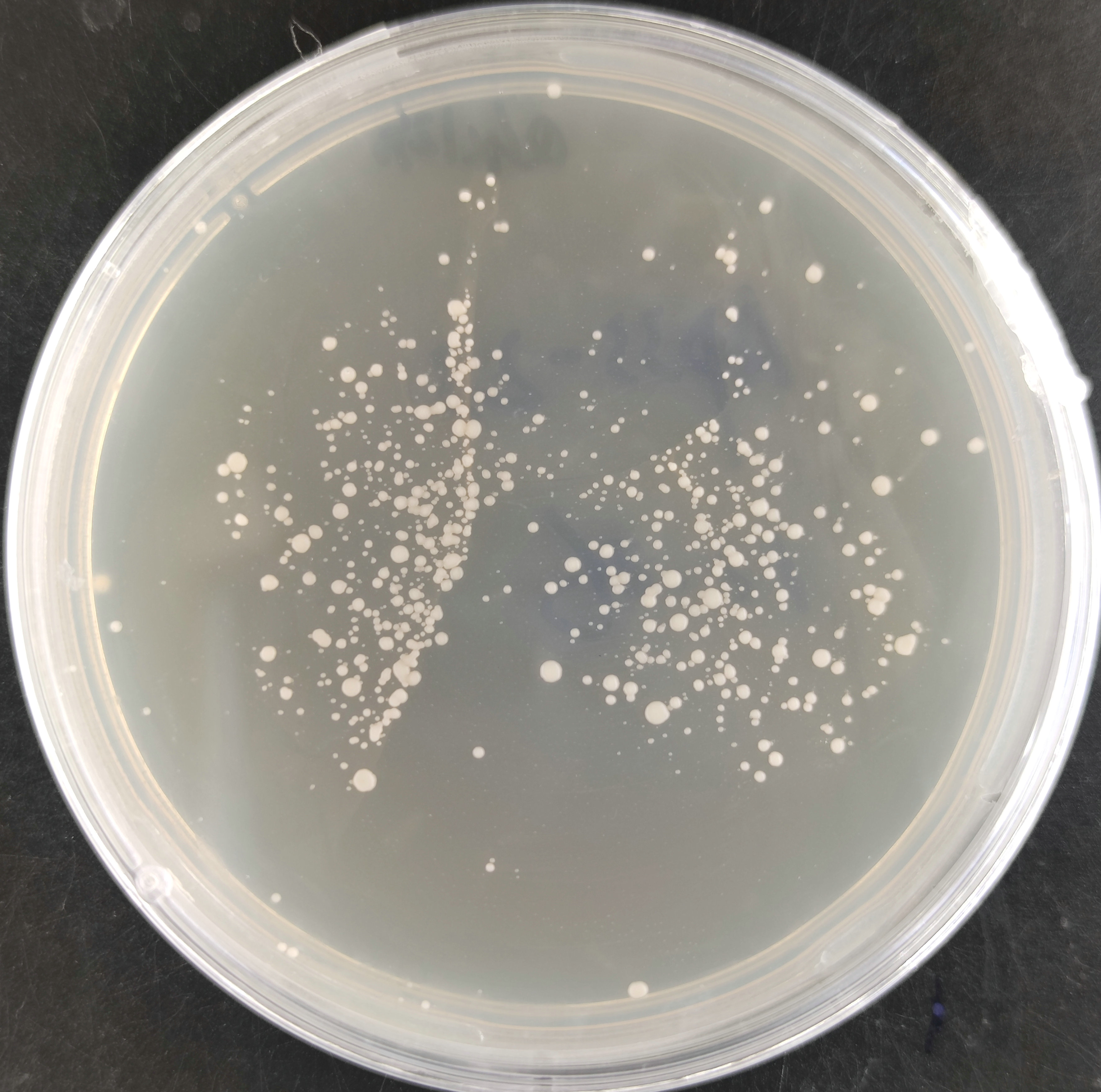

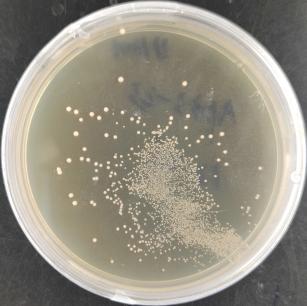

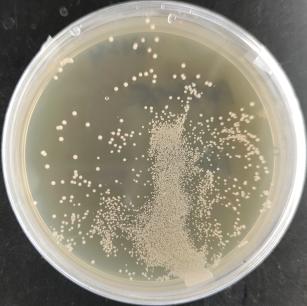


**AP33-23**

**AP33-25**


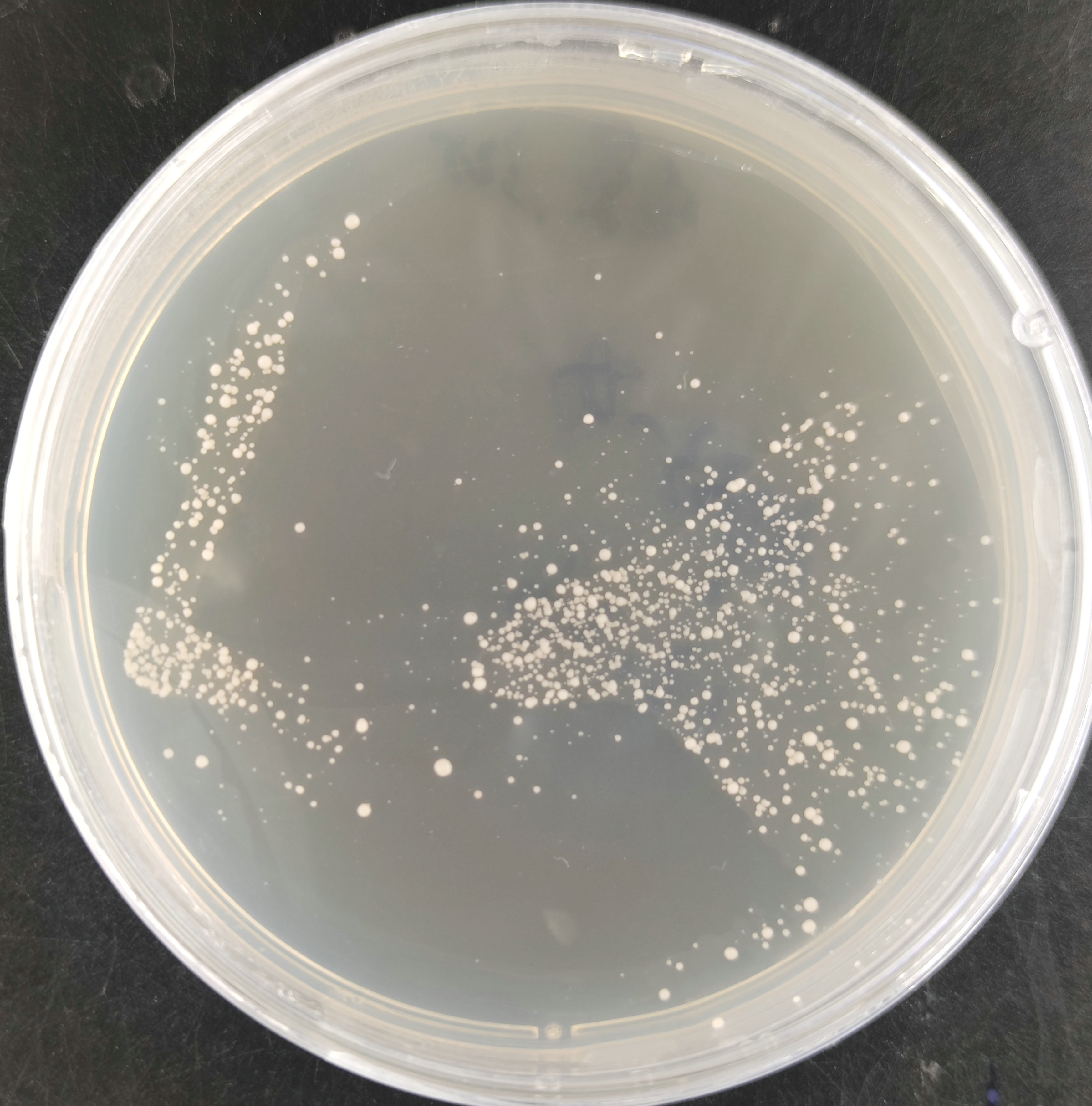

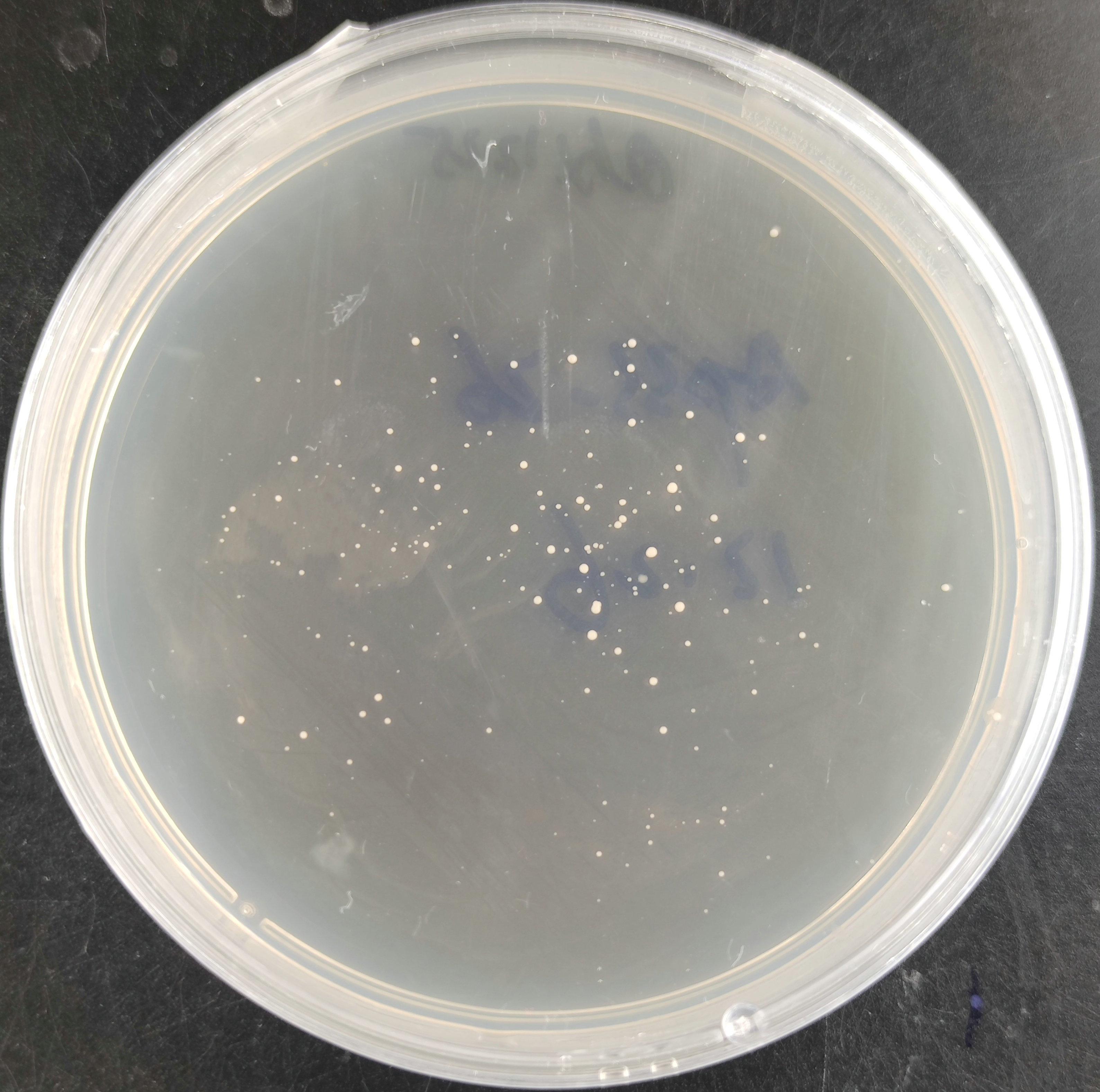

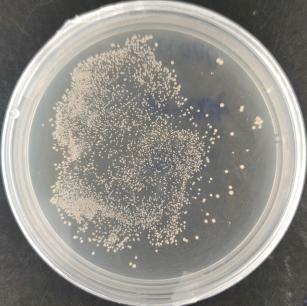

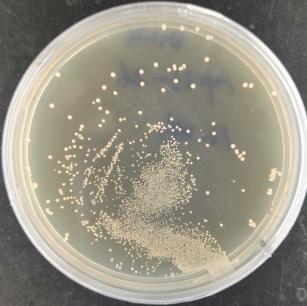


**AP33-28**

**AP33-26**


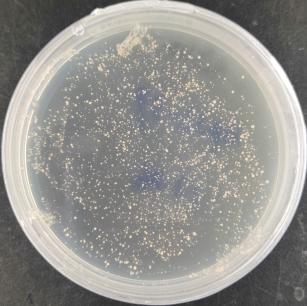

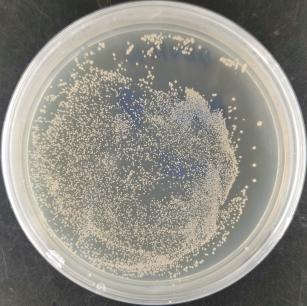

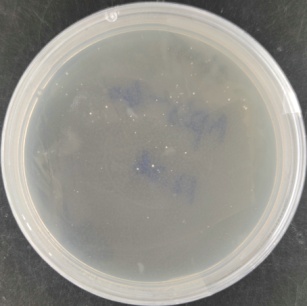

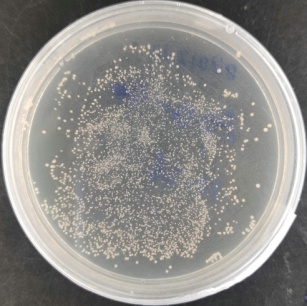


**AP33-39**

**AP33-40**


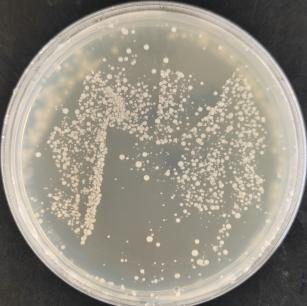

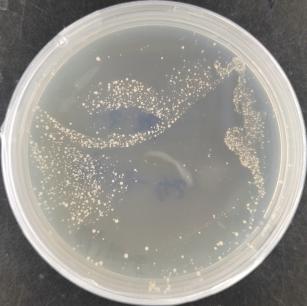

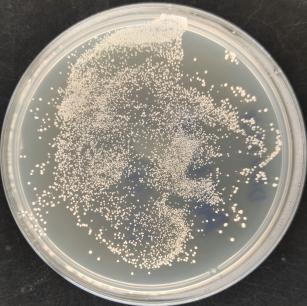

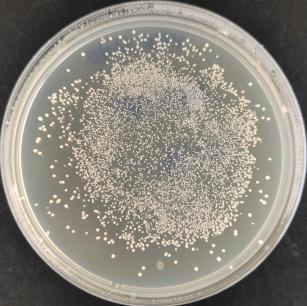


**AP33-42**

**AP33-46**

Figure Legend

The screened molecules were co-transformed into yeast cells with bait TvAP33 to verify the interaction, , respectively.
