# Supplementary material for: The interaction between adhesion protein 33 (TvAP33) and BNIP3 mediates the adhesion and pathogenicity of Trichomonas vaginalis to host cells
Source: Parasit Vectors. 2023 Jun 21;16:210. doi: 10.1186/s13071-023-05798-x (PMC10286359; doi:10.1186/s13071-023-05798-x)
Supplement: Supplementary file 7 — Additional file 7: Figure S7. The 3 alternative siRNAs. [file 13071_2023_5798_MOESM7_ESM.docx]

Additional 7

Table The 3 alternative siRNAs

| Name | siRNA Sequence (5'→3') |
| --- | --- |
| BNIP3-siRNA1 sense | GAACUGCACUUCAGCAAUAAUTT |
| BNIP3- siRNA1 antisense | AUUAUUGCUGAAGUGCAGUUCTT |
| BNIP3-siRNA2 sense | GCCUCGGUUUCUAUUUAUAAUTT |
| BNIP3-siRNA2 antisense | AUUAUAAAUAGAAACCGAGGCTT |
| BNIP3-siRNA3 sense | GCCACGUCACUUGUGUUUAUUTT |
| BNIP3-siRNA3 antisense | AAUAAACACAAGUGACGUGGCTT |

Note: BNIP3-siRNA2 was selected to knock down the expression of BNIP3 in VK2/E6E7 cells
