# Supplementary material for: The interaction between adhesion protein 33 (TvAP33) and BNIP3 mediates the adhesion and pathogenicity of Trichomonas vaginalis to host cells
Source: Parasit Vectors. 2023 Jun 21;16:210. doi: 10.1186/s13071-023-05798-x (PMC10286359; doi:10.1186/s13071-023-05798-x)
Supplement: Supplementary file 9 — Additional file 9: Figure S9. Optimization of the transfection conditions by transfecting siRNA labeled with fluorescent dye to VK2/E6E7. A Screening the optimal duration for transfecting siRNA into VK2/E6E7. A1, 6 h; A2, 12 h; A3, 24 h. B Screening the optimal siRNA concentration for transfecting siRNA into VK2/E6E7. B1, 50 nM; B2, 100 nM; B2, 150 nM. [file 13071_2023_5798_MOESM9_ESM.docx]

Additional 9

Figure


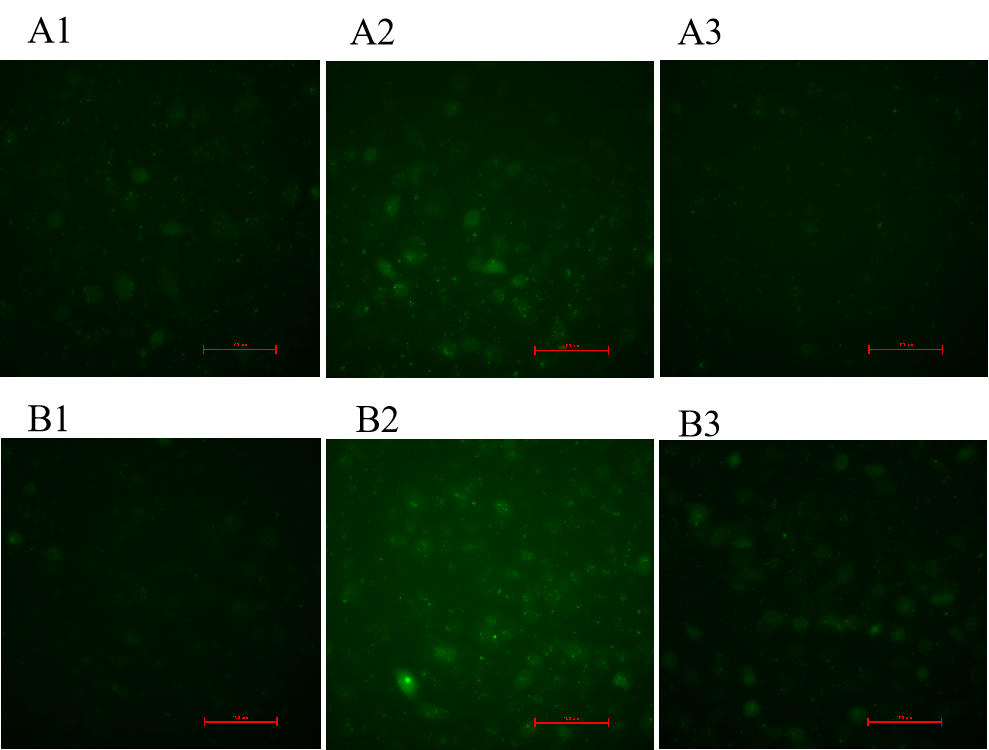


Figure Legend

Optimize the transfection conditions by transfecting siRNA labeled with fluorescent dye to VK2/E6E7. A: Screening the optimal duration for transfecting siRNA into VK2/E6E7. A1: 6 h. A2: 12 h. A3: 24 h. B: Screening the optimal siRNA concentration for transfecting siRNA into VK2/E6E7. B1: 50 nM. B2: 100 nM. B2: 150 nM.
