# Supplementary material for: The interaction between adhesion protein 33 (TvAP33) and BNIP3 mediates the adhesion and pathogenicity of Trichomonas vaginalis to host cells
Source: Parasit Vectors. 2023 Jun 21;16:210. doi: 10.1186/s13071-023-05798-x (PMC10286359; doi:10.1186/s13071-023-05798-x)
Supplement: Supplementary file 10 — Additional file 10: Figure S10. Observation of T. vaginalis adherence to VK2/E6E7 cells under the fluorescence microscope (40×). The trophozoites were stained with red fluorescence by CytoTraceTM Orange. [file 13071_2023_5798_MOESM10_ESM.docx]

Additional 10

Figure


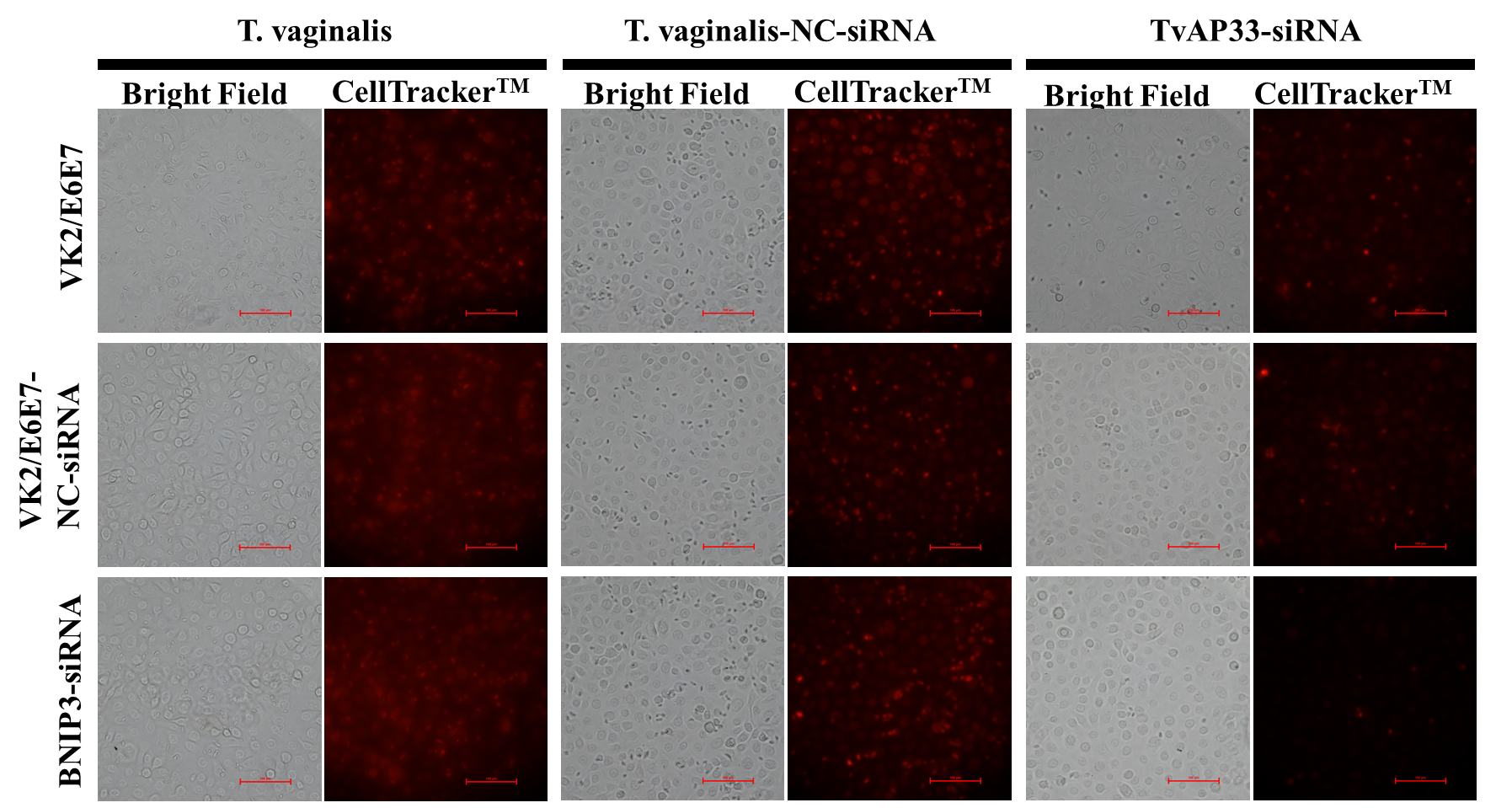


Figure Legend

Observation of *T. vaginalis* adherence to VK2/E6E7 cells under the fluorescence microscope (40×). The trophozoites were stained with red fluorescence by CytoTrace^TM^ Orange.
