# Supplementary material for: The interaction between adhesion protein 33 (TvAP33) and BNIP3 mediates the adhesion and pathogenicity of Trichomonas vaginalis to host cells
Source: Parasit Vectors. 2023 Jun 21;16:210. doi: 10.1186/s13071-023-05798-x (PMC10286359; doi:10.1186/s13071-023-05798-x)
Supplement: Supplementary file 11 — Additional file 11: Figure S11. Observation of VK2/E6E7 cells viability under the microscope (40×). These dead cells were stained with blue by trypan blue. A VK2/E6E7 cells, B T. vaginalis + VK2/E6E7 cells, C T. vaginalis + VK2/E6E7-NC-siRNA cells, D T. vaginalis + VK2/E6E7-BNIP3-siRNA cells, E T. vaginalis-NC-siRNA + VK2/E6E7 cells, F T. vaginalis-NC-siRNA + VK2/E6E7-NC-siRNA cells, G T. vaginalis-NC-siRNA + VK2/E6E7-BNIP3-siRNA cells, H T. vaginalis-TvAP33-siRNA + VK2/E6E7 cells, I T. vaginalis-TvAP33-siRNA + VK2/E6E7-NC-siRNA cells, J T. vaginalis-TvAP33-siRNA + VK2/E6E7-BNIP3-siRNA cells. [file 13071_2023_5798_MOESM11_ESM.docx]

Additional 11

Figure


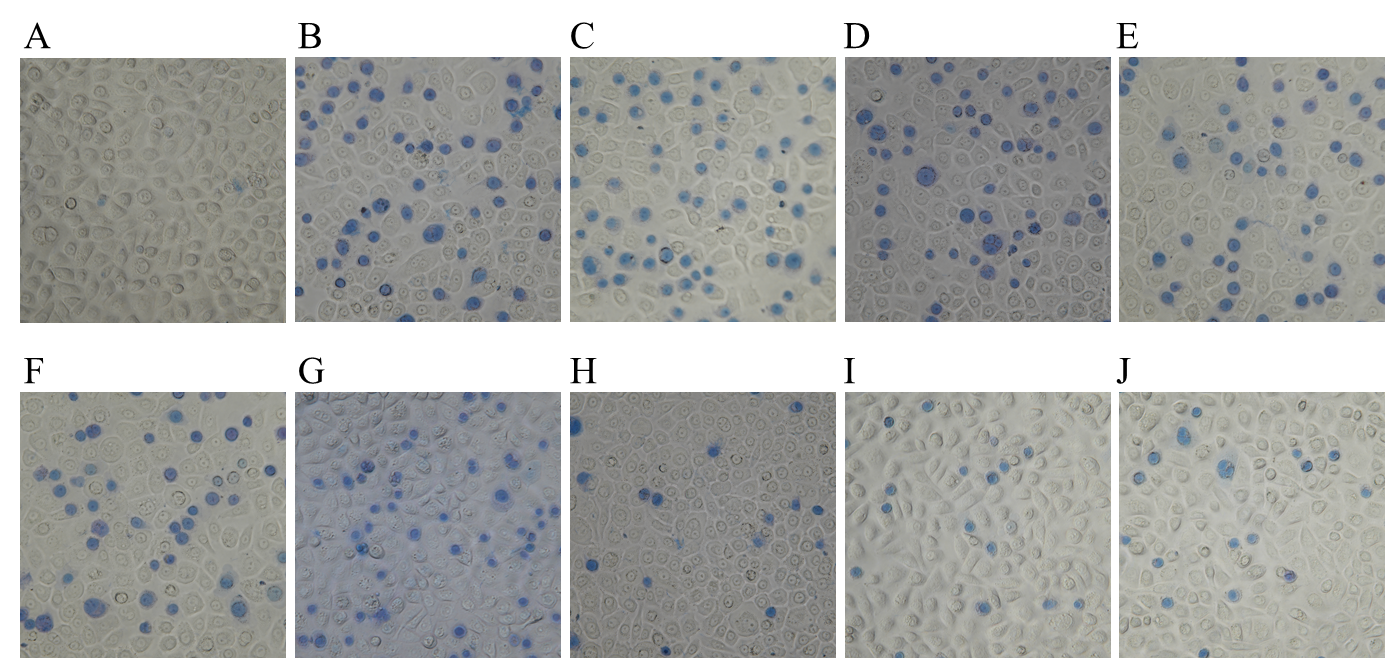


Figure Legend

Observation of VK2/E6E7 cells viability under the microscope (40×). These dead cells were stained with blue by trypan blue. A: VK2/E6E7 cells. B: *T. vaginalis +* VK2/E6E7 cells. C: *T. vaginalis +* VK2/E6E7-NC-siRNA cells. D: *T. vaginalis +* VK2/E6E7-BNIP3-siRNA cells. E: *T. vaginalis-*NC-siRNA *+* VK2/E6E7 cells. F: *T. vaginalis-*NC-siRNA *+* VK2/E6E7-NC-siRNA cells. G: *T. vaginalis-*NC-siRNA *+* VK2/E6E7-BNIP3-siRNA cells. H: *T. vaginalis-*TvAP33-siRNA *+* VK2/E6E7 cells. I: *T. vaginalis-*TvAP33-siRNA *+* VK2/E6E7-NC-siRNA cells. J: *T. vaginalis-*TvAP33-siRNA *+* VK2/E6E7-BNIP3-siRNA cells.
